# In Silico Characterisation and Determination of Gene Expression Levels of the *CPK* Family Under Saline Stress Conditions in *Chenopodium quinoa* Willd

**DOI:** 10.3390/ijms262110658

**Published:** 2025-11-01

**Authors:** Luz Lima-Huanca, Andrea Alvarez-Vasquez, María Valderrama-Valencia, Sandro Condori-Pacsi

**Affiliations:** Laboratorio de Recursos Genéticos y Genética Molecular, Universidad Nacional de San Agustín de Arequipa, Arequipa 04001, Peru; llima@unsa.edu.pe (L.L.-H.); aalvarezvas@unsa.edu.pe (A.A.-V.); mvalderramav@unsa.edu.pe (M.V.-V.)

**Keywords:** *Chenopodium quinoa*, CPK, salt stress, gene expression, stress tolerance

## Abstract

Quinoa (*Chenopodium quinoa* Willd.) is a highly nutritious crop known for its tolerance to salt stress; however, the molecular mechanisms underlying this trait remain poorly understood. This study aims to perform the in silico characterisation of calcium-dependent protein kinase (CPK) gene family sequences and to evaluate their expression profiles under salt stress conditions. Using bioinformatics tools, CPK family gene sequences were identified and in silico-characterised, including conserved domains, *cis*-regulatory motifs, and physicochemical properties. Experimentally, two contrasting accessions were compared: a salt-tolerant one (UNSA_VP033) and a salt-sensitive one (UNSA_VP021). Salt tolerance indices were determined during germination, gene expression levels were quantified by RT-qPCR, and antioxidant enzyme activities, along with malondialdehyde (MDA) content, were evaluated under different NaCl concentrations. Sixteen sequences with characteristic CPK family domains were identified. Promoter analysis revealed *cis*-elements associated with hormonal and stress responses. Physicochemical parameters predicted proteins of 50–60 kDa with variable isoelectric points. Experimentally, UNSA_VP033 showed the significant overexpression of *CqCPK12*, *CqCPK17*, *CqCPK20*, and *CqCPK32*, correlated with the higher antioxidant activity of superoxide dismutase (SOD) and peroxidase (POD), and lower MDA levels at 200 mM NaCl. In contrast, the sensitive accession exhibited significant reductions in gene expression and antioxidant activity. In conclusion CPK genes play a key role in the salt stress response in quinoa, particularly *CqCPK12*, *CqCPK17*, *CqCPK20*, and *CqCPK32* in the tolerant accession. These findings may contribute to the development of more salt-tolerant varieties, thereby enhancing agricultural sustainability in saline soils.

## 1. Introduction

Quinoa (*Chenopodium quinoa* Willd.), a species belonging to the Amaranthaceae family, was domesticated at least 5000 years ago in the Bolivian highlands. It is an allopolyploid 2n = 4x = 36 halophyte capable of adapting to a wide variety of agroecosystems. It is grown in coastal areas at sea level and high-altitude regions such as the plains of the Andean highlands (>3500 metres above sea level). In recent years, it has gained international attention for the high nutritional value of its seeds, which have an optimal balance of essential amino acids. It is also a rich source of fibre, lipids, carbohydrates, vitamins and minerals, and has the ability to tolerate abiotic stress [1,2], water stress, low temperatures and salinity stress, which has allowed it to adapt to diverse climatic conditions and extreme soils [3,4]. Quinoa has demonstrated a unique ability to regulate its ionic homeostasis, maintain the integrity of its cell membranes, and activate antioxidant mechanisms that allow it to survive and thrive in saline environments [5].

Soil salinity is one of the main problems negatively affecting agriculture worldwide, reducing crop production and quality. Anthropogenic activities, combined with climate change, exacerbate this abiotic stress through increased temperatures, enhanced evaporation, reduced humidity, and unsustainable land use. This problem jeopardises global food security and environmental sustainability [4,6]. Globally, it is estimated that one-fifth of cultivated land and approximately one-third of agricultural areas are affected by salinity stress. In Peru, salinisation problems have been identified in approximately 300,000 hectares (ha) of irrigated land, of which 150,000 ha have high levels of salinity [6]. In the coastal region, this problem has intensified, affecting more than 50% of irrigated land and posing a permanent threat to soil fertility and agricultural productivity [7]. It is projected that 30% of arable land will disappear in the next 25 years, and that by the middle of the 21st century, this loss will increase to 50%. [4,8,9]. Therefore, there is a need to understand the mechanisms of adaptation to salinity, which will allow the development of halotolerant crops to address the salinity crisis and cope with current and future climate change.

Halotolerant plants are adapted to grow under saline stress conditions. One of the mechanisms used depends on the concentration of free Ca^+2^ in the cytosol. Transient changes in cytosolic Ca^+2^ concentration initiate rapid signal transduction processes through the activation of phosphorylation cascades, which are detected and decoded by calcium-sensing proteins that regulate kinase activity. Calcium-regulated protein kinases belong to three families: calmodulin (CaM), which interacts with calcium/calmodulin-regulated kinases; Ca^+2^-sensing protein similar to calcineurin B (CBL), which regulates the protein that interacts with CBL kinases; and calcium-dependent protein kinases (CPK) [10,11].

CPK has a calcium sensor with a detection function and an effector domain corresponding to the response activity. It participates in developmental processes (flowering, pollen tube growth, fruit and root development), stress responses, cell division, and cell death [12]. In the biological model *Arabidopsis thaliana*, CPKs have a conserved structure comprising four functional domains: a serine/threonine kinase domain, an N-terminal variable domain, a self-inhibitory binding domain, and a regulatory domain containing four EF-hand-type calcium-binding motifs. The serine/threonine kinase domain is highly conserved, containing 11 subdomains responsible for ATP anchoring and orientation, the catalytic base responsible for the phosphotransferase reaction (PO_4_^3−^), and an ATP-binding catalytic domain [13,14,15]. The EF-hand motif has a helix-loop-helix conformation consisting of 29 residues in length, where the loop (DXDXS/N/D XXXXXE) consists of 12 residues. The protein is activated directly by binding with Ca^+2^, resulting in a conformational change [16,17,18]. The N-terminal domain generally has an N-myristylation site and an N-palmitoylation site, which are important for subcellular localisation and molecular function [19,20].

Multiple isoforms of calcium-dependent kinases (CPKs) have been identified in various plant species, including 34 isoforms in *Arabidopsis thaliana* [21,22], 31 in *Oryza sativa* [23], 41 in *Gossypium hirsutum* [24], 30 in *Populus trichocarpa* [25], 20 in *Triticum aestivum* [26], 29 in *Solanum habrochaites* [27], 40 in *Glycine max* [28], 36 in *Carya illinoinensis* [29], 17 in *Ananas comosus* [30], and 27 in *Medicago sativa* [31]. CPK genes have been described that play key roles in regulating plant responses to salt stress, such as in *A. thaliana*, where AtCPK8 activates enzymatic pathways involved in the elimination of reactive oxygen species (ROS) [22], and in *O. sativa*, an OsCPK12 regulates the expression of ascorbate peroxidase and NADPH oxidase OsrbohI [23]. However, to date, no studies have been published on the identification or functional characterisation of CPK isoforms in *Chenopodium quinoa.*

This study focused on performing in silico characterisation of CPK proteins and determining their gene expression levels under different salt concentrations in *C. quinoa* Willd. These findings provide significant information for quinoa cultivation and its response under saline stress conditions, and contribute to the development of more resilient and sustainable varieties for maintaining food security.

## 2. Results

### 2.1. Identification of CqCPK Genes and Analysis of Domains, Conserved Motifs and Gene Structure

**Figure 1:**
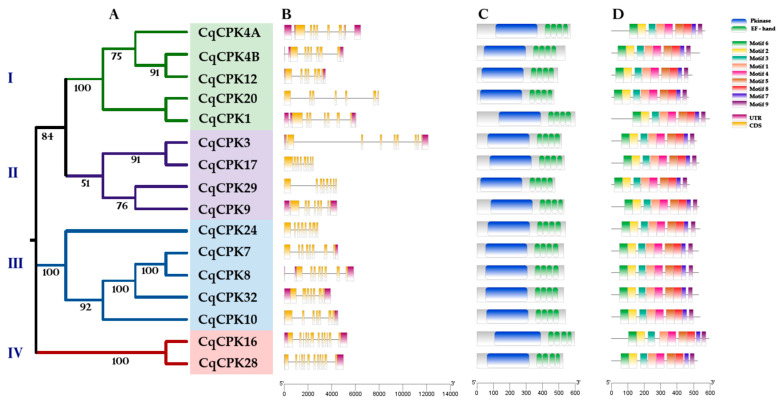
Gene structure, domains, and conserved motifs of CqCPK sequences. (**A**). Clustering of CPK proteins in *C. quinoa*. Constructed using MEGA 11 software by the Neighbour-Joining method (1000 bootstrap replicates). (**B**) Gene structure of the nucleotide sequences of CqCPKs, where the yellow rectangles correspond to exons, the black line corresponds to introns, and the purple rectangles correspond to untranslated regions (UTRs). (**C**) Conserved domains of CqCPK proteins. All proteins were analysed using CDD-NCBI to identify conserved domains. The blue bars represent serine/threonine kinase protein domains, and the green bars represent EF-hand motifs. (**D**) Structure of conserved motifs in CqCPK: the sequencing was performed in MEME. The coloured boxes represent nine conserved motifs.

After searching for CPK sequences from the *C. quinoa* genome in Phytozome and NCBI, using curated CPK sequences from *A. thaliana* as a basis, a total of 36 candidate *C. quinoa* sequences were obtained, of which only 16 sequences presented the characteristic domains of the family: a serine/threonine protein kinase domain and four Ca^+2^ -binding motifs EF-hand (Figure 1). The identified proteins were assigned the suffix Cq (*Chenopodium quinoa*), followed by the name of the group (CPK) and finally an Arabic numeral according to their registered number, followed by a letter in sequence (A, B). These proteins were identified using the information available from CDD-NCBI, and the number and position of the domains of each CqCPK protein were identified.

Figure 1A shows that the protein sequences of CqCPKs have four defined groups (I, II, III, and IV). Figure 1B presents the structural analysis of exons-introns, which shows the variation in the number of exons (six to twelve) and introns (six to eleven) among the CqCPK sequences analysed. Although the size of the exons is relatively conserved, slight variations are observed that could be associated with specific functional adaptations. Likewise, some genes, such as *CqCPK3* and *CqCPK20*, have considerably longer introns, even among members of the same phylogenetic group, suggesting intronic expansion events or differential regulation mechanisms. Group I has seven conserved exons, group II has members with eight exons and one with nine, group III has two members with seven exons and three with eight exons, presenting a mixed structural composition, and group IV has two members with twelve exons.

Figure 1C shows the distribution of the characteristic domains of the CPK family. The serine/threonine protein kinase domain (blue bars) has a highly similar length and location among the different proteins, suggesting strong evolutionary conservation of its catalytic function and four Ca^2+^-binding motifs. EF-hand (green boxes) show a very stable arrangement in terms of relative position and size throughout all CqCPK sequences, indicating that they belong to the CPK family, thus showing that the domains are very well conserved in all proteins. In Figure 1D, a total of 9 conserved motifs were identified using the MEME tool, where the 16 protein sequences presented similar distribution and number of motifs, 5 motifs (1, 2, 3, 4 and 6) correspond to the 11 subdomains of serine/threonine kinase. In particular, motifs 2 and 6 are located at the amino-terminal end and integrate subdomains I to V, which include the ATP binding site. On the other hand, motifs 1, 3 and 4 are located at the carboxy-terminal end and include subdomains VI to XI, related to substrate binding and phosphotransferase activity. Additionally, motifs 5, 7, 8 and 9 correspond to the four EF-hand motifs, commonly associated with calcium binding

### 2.2. Physicochemical Characterisation of CqCPK Genes

Table 1 shows the physicochemical characteristics of CqCPKs obtained using the ExPASy web server. the gene size varies between 2451 (*CqCPK17*) and 12143 (*CqCPK3*) bp, and the number of amino acids varies between 470 (*CqCPK20*) and 599 (*CqCPK1*) aa. The molecular weights vary between 52,599 (*CqCPK20*) and 66,797 (*CqCPK16*) kDa, and their isoelectric point (pI) is between 5.02 (*CqCPK20*) and 9.15. (*CqCPK16)* To indicate structural stability, the aliphatic index of the sequences was determined, obtaining values ranging from 76.85 (*CqCPK17*) to 92.49 (*CqCPK20*).

The prediction of myristoylation and palmitoylation sites showed that four isoforms contain these sites, six isoforms contain only palmitoylation sites, and six isoforms do not contain any predicted post-translational modification sites at their N-termini. The prediction of subcellular localisation indicates that CqCPKs can be located in different subcellular compartments; all CqCPKs are located in the cytoplasm, and some proteins can also be found in the nucleus and mitochondria. Analysis of the N-terminal revealed conserved acylation motifs such as MGNTCVRP, MGNCCGSP, and MGNCNACI, reinforcing the possibility of lipid-dependent regulation. A glycine in the second position, a key feature for N-myristoylation, was identified in several proteins, while cysteine residues in adjacent positions could facilitate S-palmitoylation.

### 2.3. Sequence Alignment and Phylogenetic Analysis

**Figure 2:**
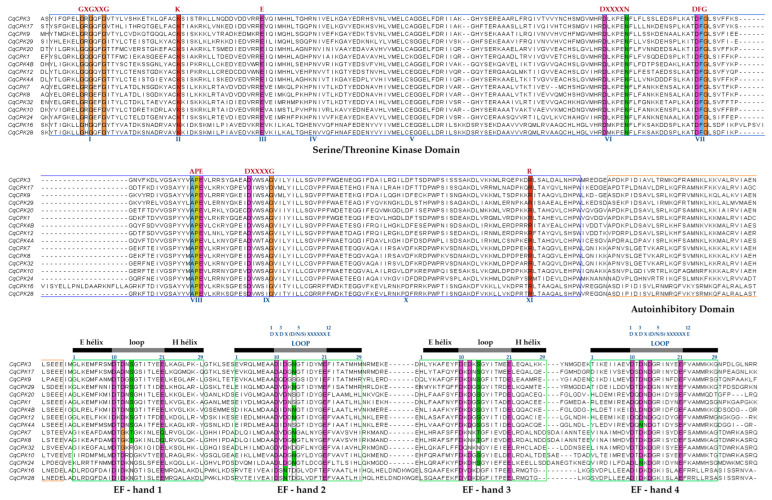
Alignment of multiple amino acid sequences of CqCPKs. Amino acid residues conserved across all sequences are highlighted in different colours, indicating conserved domains corresponding to a serine/threonine protein kinase domain (blue box), a self-inhibitory domain (orange box) and four EF-hand Ca^2+^ binding motifs (green box). The relative positions of the amino acids are shown at the bottom, while at the top, the black and grey bars represent the predicted secondary structures: alpha helices and loops, respectively. The regions marked as “LOOP” indicate the typical sequence of 12 residues that form the Ca^2+^ coordination site within the EF-hand motifs.

Figure 2 shows the sequence alignment in Clustal Omega, where it can be seen that all *C. quinoa* protein sequences contain a serine/threonine protein kinase domain (blue box), an autoinhibitory domain (orange box) and four Ca^+2^ EF-hand binding motifs that define them as CPK family proteins (grey and black bars). In the case of the EF-hand motif, it has a helix-loop-helix conformation consisting of 29 residues in length, where the loop (DXDX S/N/D XXXXXE) consists of 12 residues located at positions 10 to 21. Position 12 of the loop is essential for the stability of the protein–Ca^+2^ complex. The CqCPK7 and CqCPK8 isoforms in EF-hand 1 show a change in the glutamic acid amino acid at position 12 of the loop to glutamine 12.

**Figure 3:**
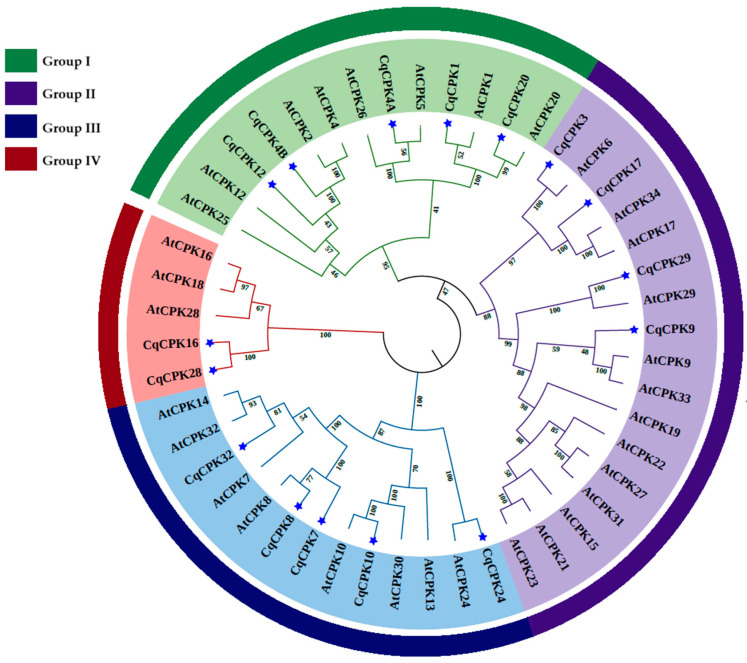
Phylogenetic tree of CPK genes in *Chenopodium quinoa* and *Arabidopsis thaliana*, constructed in MEGA X. We used the Neighbour-Joining method and Jones–Taylor–Thornton (JTT) substitution model, with gamma distribution (G), displaying a bootstrap value of 1000. The sequences are grouped into four subgroups: group I (green), group II (lilac), group III (green) and group IV (red). The blue stars represent the CPKs of *C. quinoa*.

All 16 CPK proteins from quinoa (*C. quinoa*) and 32 proteins from Arabidopsis (*A. thaliana*) were aligned to generate a phylogenetic tree using the Neighbour-Joining method and Jones–Taylor–Thornton (JTT) substitution model, with gamma (G) distribution, performed in MEGA X. Figure 3 shows the phylogenetic tree where CqCPKs form four groups or subfamilies I, II, III, IV based on their similarity. In each of these groups, CPK genes are more closely related to each other, suggesting that they may share similar or complementary functions in different species. In *C. quinoa*, group I consists of 5 CqCPK isoforms (1, 4A, 4B, 12 and 20), group II has 4 CqCPKs (3, 9, 17 and 29), group III consists of 5 CqCPK isoforms (7, 8, 10, 24, and 32), and group IV consists of 2 CqCPK isoforms (16 and 28).

### 2.4. Analysis of the Promoter Regions of Elements Acting in cis on CqCPK Genes

**Figure 4:**
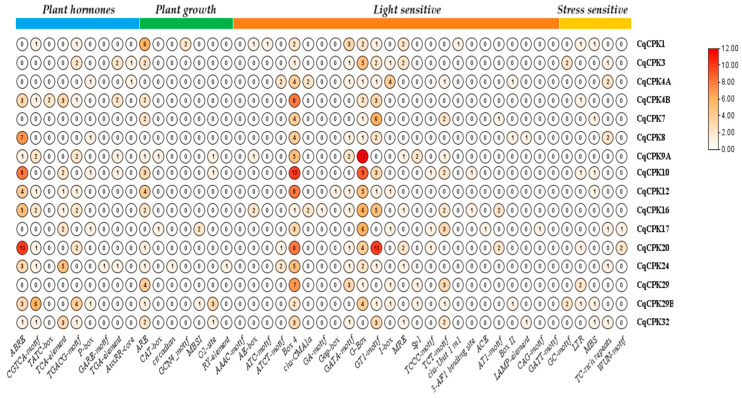
Analysis of *cis*-elements in the promoters of CqCPK proteins. The coloured bars at the top indicate the categorised groups. The frequency of each *cis*-element is shown by a number inside the circle. The colour intensity of the circle is proportional to the score, and the corresponding relationship between numbers and colours is shown by a colour scale in the right-hand panel.

In order to understand the possible functions of CqCPK genes, the promoter region 2000 bp upstream of the start codon (ATG) of CqCPK genes was analysed using the Plant-CARE online server. Figure 4 shows the *cis*-acting elements. A total of 45 elements were identified (Table A1), which were divided into four groups: (1) response to plant hormones, (2) response to light, (3) plant growth, and (4) response to stress.

The plant hormone group contains five elements, which are involved in the response to abscisic acid (ABRE), response to methyl jasmonate (MeJA) (CGTCA-motif and TGACG-motif), response to gibberellins (TATC-box, P-box and GARE-motif), salicylic acid response (TCA-element), and auxin response (TGA-element and AuxRR-core). Genes such as *CqCPK8*, *CqCPK10* and *CqCPK20* had 7, 8 and 10 copies of ABRE motifs, respectively, suggesting possible intensive regulation by ABA under saline stress conditions.

The light response group contains 24 elements, constituting the group with the highest number of identified motifs. In particular, the genes *CqCPK9*, *CqCPK10*, *CqCPK16*, and *CqCPK20* had the highest number of regulatory elements, with 24, 26, 24, and 29, respectively. The most frequently occurring motifs were Box 4, GT1-motif and I-box. The plant growth group contains seven elements, which are involved in endosperm expression (GCN4_motif), anaerobic induction (ARE), meristem expression (CAT-box), circadian control (circadian), endosperm expression (GCN4_motif), regulation of flavonoid biosynthetic genes (MBSI), regulation of zein metabolism (O2-site) and regulation involved in seed specificity (RY-element).

Analysis of *cis*-regulatory elements revealed a widespread presence of motifs related to the abiotic and biotic stress response of *CqCPK* genes, suggesting a key role in the adaptation of *C. quinoa* to adverse conditions. *CqCPK4B* had the highest number of stress-associated elements (5 elements), followed by *CqCPK10* and *CqCPK20*, each with 4 elements. Other genes, such as *CqCPK1*, *CqCPK3*, *CqCPK12*, and *CqCPK32*, also showed a notable recurrence of these motifs, reinforcing the hypothesis that these genes could be highly induced in multiple stress contexts. These include motifs such as GC-motif (hypoxic stress), LTR (response to low temperatures), MBS (response to drought), TC-rich repeats (general response to stress), and WUN-motif (response to wounds). The cumulative presence of these motifs in certain genes suggests a possible complex regulation in the maintenance of growth and development, but also plays a decisive role in the plant’s resilience to stress conditions.

### 2.5. Evaluation of Salt Tolerance in C. quinoa

#### Germination of *C. quinoa* Seeds and Determination of Salt Stress Tolerance Indices

**Figure 5:**
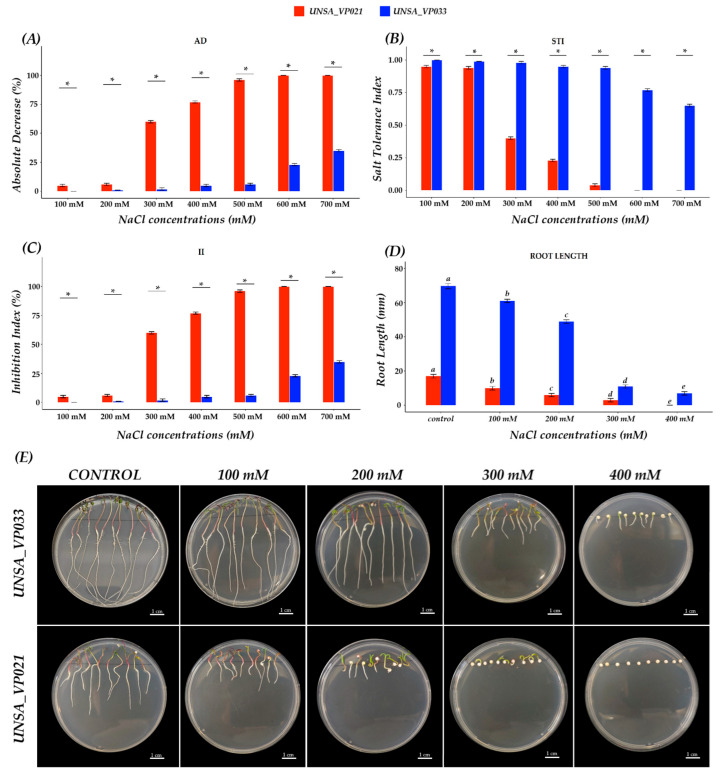
Salinity tolerance indices during germination of *C. quinoa* W. accessions UNSA_VP033 and UNSA_VP021 under different NaCl concentrations (0 mM, 100 mM, 200 mM, 300 mM, 400 mM, 500 mM, 600 mM, and 700 mM). (**A**) Absolute decrease index. (**B**) Salt tolerance index. (**C**) Inhibition index. Data were evaluated using Student’s *t*-test. Asterisks (*) indicate significant differences between groups at a significance level of *p* < 0.05. (**D**) Evaluation of root length under salinity conditions. (**E**) Root growth in MS medium supplemented with NaCl (0, 100, 200, 300, 400 mM) after 5 days of growth. Scale bars: 1 cm. Root length data were analysed using ANOVA followed by Tukey’s post hoc test (HDS). Different letters indicate significant differences between treatments (*p* < 0.05).

The effect of salinity on the germination process of quinoa seeds was evaluated at concentrations of 100, 200, 300, 400, 500, 600, and 700 mM NaCl, as shown in Figure 5A. A reduction in the absolute decrease (AD) rate of germination was observed in accessions UNSA_VP021 and UNSA_VP033, where the sensitive accession UNSA_VP021 at 300 mM showed a 59% reduction in germination, and at 400 and 500 mM, germination was reduced by 77% and 96%, respectively.

The tolerant accession UNSA_VP033 from 100 to 500 mM showed a reduction in germination between 1 and 3%, while at 600 and 700 mM, germination was reduced by 22 and 36%, respectively. Figure 5B shows the salt tolerance index (STI), which shows that accession UNSA_VP033 with 100 to 500 mM NaCl showed an STI of 99% to 97% as the salt concentration increased. At concentrations of 600 and 700 mM, the STI decreased to 74% and 78%, respectively. In accession UNSA_VP021, the STI at 100 and 200 mM remained at 98%, while at 300, 400, and 500 mM it decreased to 41%, 23%, and 4%, respectively. At 600 and 700 mM, no germination was observed.

Figure 5C shows the inhibition index (II), where accession UNSA_VP021, at 100 and 200 mM, the germination percentage was inhibited by up to 6%, at 300 mM the germination percentage was inhibited by 60%, at 400 and 500 mM, inhibition was 77% and 96%, respectively, and at 600 and 700 mM, germination inhibition was 100%. Accession UNSA_VP033 at concentrations of 100 to 500 mM, the germination inhibition index remained between 1 and 3%; at 600 and 700 mM, the inhibition percentage was 22 and 36%, respectively.

Figure 5D shows the evaluation of root length in MS medium under salinity conditions. The results showed a progressive reduction in root length as the salt concentration increased in both accessions (Figure 5D; Table A2). At 100 mM NaCl, both accessions showed a reduction in root length compared to the control. UNSA_VP021 showed a 41.2% reduction compared to the control. In contrast, UNSA_VP033 showed a 12.4% reduction compared to the control. When the concentration was increased to 200 mM, root lengths showed a reduction of 64.7% in UNSA_VP021 and 29.7% in UNSA_VP033. At 300 mM, UNSA_VP021 showed a significant decrease (*p* < 0.05), reaching an 82.4% reduction in length, and UNSA_VP033 showed an 84.2% reduction. Finally, at 400 mM NaCl, a total halt in root growth was observed in UNSA_VP021, while UNSA_VP033, although also showing a significant reduction (*p* < 0.05), maintained 10% of its initial length. These results indicate that UNSA_VP033 has a higher tolerance to salinity compared to UNSA_VP021.

### 2.6. Relative Expression of CPK Genes in C. quinoa

**Figure A1:**
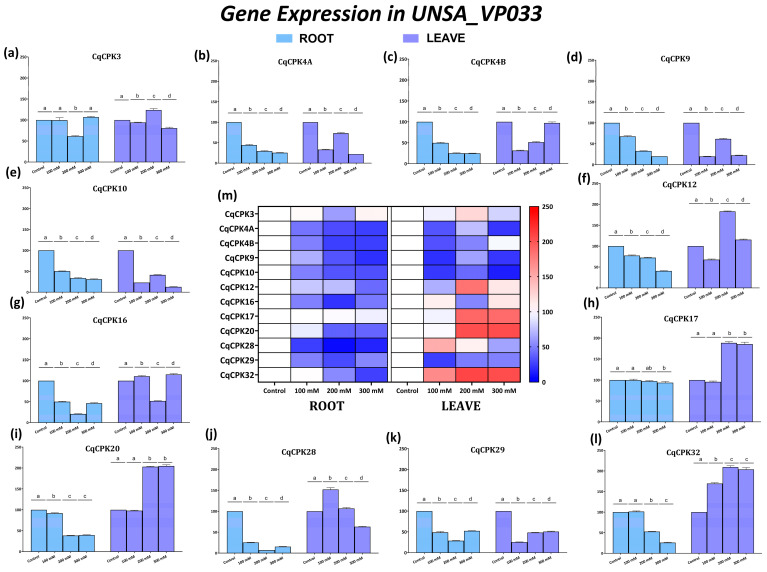
Relative expression of CPK genes in the tolerant accession UNSA_VP033 at different concentrations compared to the control. Panels (**a**–**l**) show the relative expression levels of each *CqCPK* gene, while panel (**m**) presents the heatmap summarising gene expression patterns in this accession. Data were analysed using ANOVA followed by Tukey’s post hoc test (HDS). Different letters indicate significant differences between treatments (*p* < 0.05).

For sampling for qPCR analysis, the plants were previously subjected to different salinity concentrations (0, 100, 200, 300, 400, 500, 600, and 700) mM to find the median lethal dose (LD_50_) under hydroponic conditions. It was found that accession UNSA_VP033 presented 50% mortality at 400 mM, while accession UNSA_VP021 presented 50% mortality at 300 mM. With these preliminary results, expression was evaluated at 0, 100, and 200 mM for both accessions, while accession UNSA_VP033 was evaluated up to 300 mM (Figure A1).

**Figure 6:**
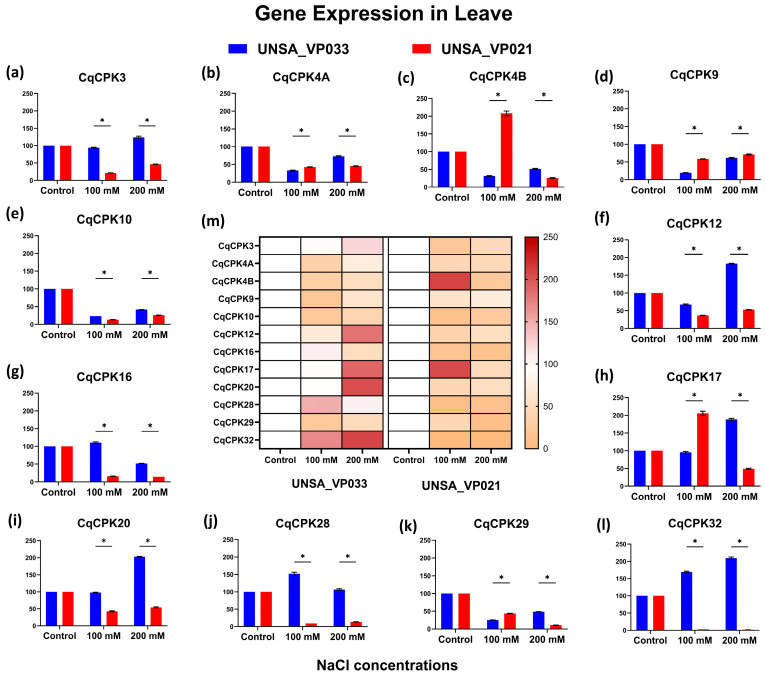
Relative gene expression of the CqCPK family in leaves of *C. quinoa* accessions UNSA_VP033 and UNSA_VP021 under saline stress conditions. Subfigures (**a**–**l**) show the relative expression levels of each CqCPK gene, while subfigure (**m**) presents the heatmap summarising gene expression patterns in both accessions. The vertical bars represent the mean ± standard deviation (SD) of three biological replicates. The control condition was used as the reference value (100%), so no error bars are shown for this group. Asterisks (*) indicate significant differences between groups at a significance level of *p* < 0.05.

Figure 6 shows the expression levels in quinoa leaves of accessions UNSA_VP033 and UNSA_VP021 under different concentrations of sodium chloride. The heat map (central figure) and bars show that the different CqCPK genes presented differential variation in expression levels compared to the control. In accession UNSA_VP033, at 100 mM, the *CqCPK32* and *CqCPK28* genes showed relative expression of 169.38 and 152.38, respectively. at 200 mM, the genes *CqCPK32*, *CqCPK20*, *CqCPK17*, and *CqCPK12* showed expression levels of 209.29, 202.93, 188.28, and 182.85, respectively, values significantly higher than the control (*p* < 0.05), and at 300 mM (results shown in Figure A1), the genes *CqCPK20*, *CqCPK32*, and *CqCPK17* showed expression levels of 205.06, 203.7, and 185.2, respectively, values significantly higher than the control (*p* < 0.05). The genes *CqCPK4A*, *CqCPK9*, *CqCPK10*, *CqCPK4B* and *CqCPK29* showed expression below the control, and these values were significantly different from the control (*p* < 0.05) at 100 and 200 mM.

In accession UNSA_VP021, at 100 mM, the *CqCPK4B* and *CqCPK17* genes showed expression of 207.86 and 205.96, respectively, values significantly higher than the control (*p* < 0.05); at 200 mM, all levels of *CqCPK* gene expression were below the control.

**Figure 7:**
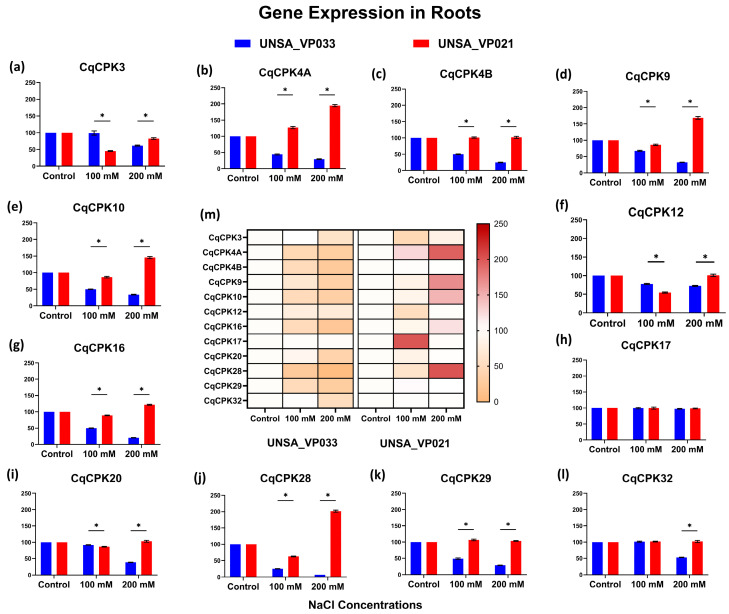
Relative gene expression of the *CqCPK* family in roots of *C. quinoa* accessions UNSA_VP033 and UNSA_VP021 under saline stress conditions. Subfigures (**a**–**l**) show the relative expression levels of each *CqCPK* gene, while subfigure (**m**) presents the heatmap summarising gene expression patterns in both accessions. The vertical bars represent the mean ± standard deviation (SD) of three biological replicates. The control condition was used as the reference value (100%), so no error bars are shown for this group. Asterisks (*) indicate significant differences between groups at a significance level of *p* < 0.05.

Figure 7 shows the expression levels in quinoa roots of accessions UNSA_VP033 and UNSA_VP021 under different salinity concentrations. The heat map (central figure) and bars show that the different *CqCPK* genes presented differential variation in expression levels compared to the control. In accession UNSA_VP021, the *CqCPK4A* gene showed higher relative expression, reaching a value of 127 at 100 mM, which was significant compared to the control. At 200 mM, the *CqCPK28*, *CqCPK4A*, *CqCPK9*, *CqCPK10*, and *CqCPK16* presented expression levels of 201.43, 194.50, 168.59, 145.36, and 121.61, respectively, values significantly higher than the control (*p* < 0.05). In contrast, the genes *CqCPK3*, *CqCPK12*, *CqCPK20*, *CqCPK4B*, and *CqCPK29* showed a significant decrease (*p* < 0.05) in expression compared to the control under low saline stress conditions.

In accession UNSA_VP033, all genes evaluated exhibited lower relative expression, with the genes *CqCPK28*, *CqCPK16*, *CqCPK4B*, *CqCPK4A*, and *CqCPK9*, *CqCPK10*, and *CqCPK20* had expression levels of 7.17, 20.8, 25.09, 29.12, 32.57, 33.53, and 38.28, respectively, values significantly lower than the control (*p* < 0.05).

### 2.7. Enzymatic Analysis and Malondialdehyde Production

**Figure 8:**
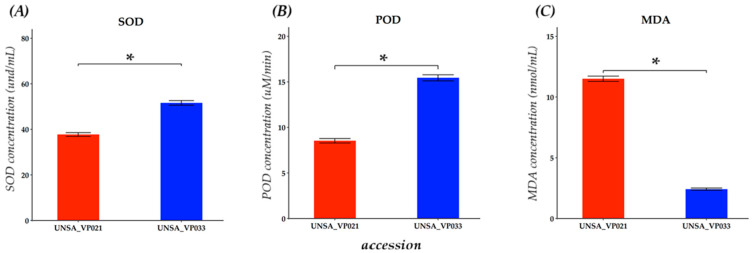
Analysis of MDA content and antioxidant enzymes SOD and POD in leaves of *C. quinoa* Willd accessions UNSA_VP033 and UNSA_VP021 subjected to 200 mM NaCl. (**A**) Quantification of superoxide dismutase activity in und/mL. (**B**) Quantification of peroxidase activity expressed in µM/min (**C**) Malondialdehyde content expressed in nmol/mL. Data were evaluated using Student’s *t*-test. Asterisks (*) indicate significant differences between groups at a significance level of *p* < 0.05.

The effects of salt stress on indicators of oxidative damage and antioxidant activity were evaluated in leaves of accessions UNSA_VP021 and UNSA_VP033, treated for five days with 200 mM NaCl (Figure 8). This concentration was selected based on the determination of LD_50_, where it was observed that 200 mM represents a sublethal condition suitable for inducing physiological responses without causing significant mortality in the accessions evaluated. The results presented below correspond to differential values (Δ) calculated as the difference between the treatment with 200 mM NaCl and the corresponding control for each accession, with the aim of representing the net effect of salt stress on enzyme activity and lipid peroxidation in quinoa leaves.

In Figure 8A, accession UNSA_VP033 showed significantly higher SOD activity compared to UNSA_VP021, reaching 51.62 U/mL versus 37.73 U/mL, respectively (*p* < 0.05). This difference suggests that UNSA_VP033 has a greater capacity to activate this antioxidant pathway under saline stress conditions.

Similarly, in Figure 8B, peroxidase (POD) activity was also significantly higher (*p* < 0.05) in UNSA_VP033, with an activity of 15.06 µM/min, while UNSA_VP021 had an activity of 8.15 µM/min. These results reinforce the idea of a more efficient antioxidant response in UNSA_VP033.

In contrast, Figure 8C shows that malondialdehyde (MDA) levels, a marker of lipid peroxidation, were considerably higher in UNSA_VP021 with 11.50 nmol/mL compared to UNSA_VP033, which had a concentration of 2.42 nmol/mL, indicating greater oxidative damage in the less tolerant accession (UNSA_VP021).

## 3. Discussion

### 3.1. Identification of CPK Family Genes C. quinoa

Calcium-dependent protein kinases (CPKs) constitute one of the main families of kinases in plants, playing crucial roles in physiological processes and responses to multiple types of stress [17,30]. In this study, 16 *CqCPK* genes were identified. The conserved grouping of proteins observed in Figure 3 suggests that the subgroups maintain close evolutionary relationships and possibly share similar biological functions. Phylogenetic analysis grouped these genes into four subgroups (I, II, III, and IV) (Figure 1A), consistent with CPK classifications in other species, such as *Arabidopsis thaliana*, *Oryza sativa*, *A. comosus*, and *Gossypium hirsutum* [30,32,33,34]. All *CqCPKs* in quinoa had characteristic structural domains (Figure 1B), such as a variable N-terminal domain, an autoinhibitory domain, a phosphorylation kinase domain, and four EF-hand motifs arranged in two pairs [35]. Which optimises their calcium-binding capacity and structural stability in their active form. This structural conservation has been observed in other studies in species such as *O. sativa*, *Fragaria × ananassa*, and *A. comosus* [30,33,36].

Analysis of conserved MEME motifs (Figure 1C) revealed that CqCPKs contain nine main motifs that show high conservation in organisation and number in all CqCPKs. Similar motif organisation was found in research on *Glycyrrhiza uralensis* [37] and *Chorchorus capsularis* [38]. Sequence alignment analysis (Figure 2) identified highly conserved residues within the functional domains, supporting their evolutionary and functional importance. In agreement with previous studies by Hanks [14] and Takahashi & Ito, it was observed that serine/threonine kinase contains eleven typically conserved subdomains. The amino-terminal end is essential for ATP binding, containing the conserved sequence GXGXXG in subdomain I, which is a glycine-rich loop whose function is phosphate binding, while lysine (K) in subdomain II and glutamic acid (E) in subdomain III participate in the stabilisation and orientation of ATP. At the carboxy-terminal end, the conserved sequence DXXXXN was identified in subdomain VIb, the catalytic ATP-binding domain, while the DFG sequence in subdomain VII plays a structural role in the coordination of magnesium ions. In addition, conserved APE motifs have been detected in subdomain VIII, DXXXXG in subdomain IX, and a highly conserved arginine (R) in subdomain XI. These results are consistent with the reports of Nolen et al. [39]. Furthermore, according to Wang et al. [40], the conservation of these residues suggests strong evolutionary pressure to maintain the functional and structural integrity of the kinase domain.

EF-hand motifs, whose main function is to coordinate calcium ion (Ca^+2^) binding, have a helix-loop-helix conformation (approximately 29 aa), with the loop (DXDX S/N/D XXXXXE) typically consisting of 12 aa located at positions 10 to 21, as previously described by Liese & Romeis [11]. his region provides flexibility for calcium ion binding, thanks to the presence of glutamic acid (E), a negatively charged amino acid, at position 12 of the loop. This is a bidentate ligand that provides two oxygen atoms, necessary for complete calcium stabilisation, a characteristic consistently reported in various EF-hand proteins by Kundu et al. [18], McCormack et al. [41], and Grabarek [42]. The conservation of the EF-hand motif is also observed in species such as *G. uralensis* [37], *Zea mays* [40], and *C. capsularis* [38]. In addition, variations were identified in the sequences of the EF-hand motif of *CqCPK7* and *CqCPK8*, where glutamic acid (E) at position 12 of the loop was replaced by glutamine (Q). This change suggests a possible alteration in calcium ion affinity, which could result in decreased sensitivity or a partial loss of calcium uptake and a consequent conformational change in the entire CPK protein. This finding is similar to that reported in *A. thaliana* by Liese & Romeis [11], where EF-hand 1 of *AtCPK23* is degenerate due to the substitution of glutamic acid (E) for glutamine (Q), which has been associated with a decrease in affinity for calcium ions and, therefore, with a reduced functional capacity in calcium uptake and response to calcium signals.

Structurally, *CqCPK* genes show variation in the number of exons (Figure 1D). Consistent patterns were observed within each group: members of group I had seven exons, while those in group II had eight, with the exception of *CqCPK3*, which has nine. In group III, seven and eight exons were observed, while the two members of group IV retained 12 exons, suggesting that this latter group has been under greater evolutionary pressure to maintain its structure. Studies in *A. thaliana* [32], *A. comosus* [30], *Vittis* spp. [43], *G. hirsutum* [34] show similar patterns. The reduction in the number of exons in certain CqCPK genes could be explained by genomic deletion events, structural rearrangements, or selective pressures that favour faster or more efficient transcription under adverse conditions such as salinity. This hypothesis is supported by recent studies by Hernández-Urrieta et al. [44], that show how environmental stress modulates gene structure to favour adaptive responses. In particular, it has been reported that salinity and other factors induce changes in alternative splicing, affecting the inclusion or exclusion of exons and generating more functional isoforms under adverse conditions. Comparative studies on MADS-box genes, such as those by Yu et al. [45], have shown that structural changes in exon-intron organisation are frequent and may be linked to functional specialisation, suggesting an evolutionary background that explains the structural variability present in *CqCPK* genes.

The comparative analysis between *C. quinoa* and *A. thaliana* (Figure 3) showed that *CPK* genes in both species are grouped into four subgroups (I–IV), a pattern consistent with that reported in other model species such as *Vittis* spp. [19,43] *O. sativa* [33] and *A. comosus* [30], suggesting strong evolutionary pressure to maintain similar functions within each subfamily in different species. The high similarity in the *CPK* genes of *A. thaliana* and *C. quinoa* indicates that they are closely related to each other, suggesting that they may retain similar or complementary functions, as they maintain greater conservation in key functional regions, supporting their possible involvement in specific responses, their subcellular localisation, or their participation in specific signalling pathways, as reported by Li, et al. [46] where phylogenetically related *CPK* genes can be located in the same subcellular compartments or participate in common responses to biotic or abiotic stimuli.

The physicochemical characterisation (Table 1) reveals sizes ranging from 470 to amino acids and estimated molecular weights between 52,599 and 66,797 kDa. These values are within the range reported for CPK proteins in other species such as *Triticum aestivum* [47] and *G. hirsutum* [34], suggesting general structural conservation between species. The isoelectric point (pI) ranges from 5.02 to 9.15, similar to that observed in *A. comosus* [30] and *G. uralensis* [37]. The aliphatic index, which is related to the thermal stability of proteins, ranged from 76.85 to 92.49, suggesting high stability, in agreement with that reported in wheat by Liu et al. [47].

Most CqCPK proteins, except *CqCPK3*, *CqCPK4B*, *CqCPK12*, *CqCPK20*, *CqCPK28*, and *CqCPK29*, undergo post-translational modifications, specifically N-myristoylation and S-palmitoylation sites, according to Cheng et al. [32]. Post-translational modifications are essential for their subcellular localisation and function. In *O. sativa* [48], *OsCPK19* has been shown to possess both types of modifications, which determine its association with the cell membrane. In accordance with this model, in the present study, the prediction of myristoylation and palmitoylation sites showed that four isoforms (*CqCPK9*, *CqCPK10*, *CqCPK16*, and *CqCPK24*) simultaneously contain myristoylation and palmitoylation sites, while six (*CqCPK1*, *CqCPK4A*, *CqCPK7*, *CqCPK8*, *CqCPK17*, *CqCPK32*) have only palmitoylation sites. This pattern is consistent with what has been reported in species such as *Fragaria* [36] and *Vitis* spp. [43]. However, the prediction of subcellular localisation suggests that CPKs in quinoa, despite undergoing these modifications, are not restricted solely to the plasma membrane. On the contrary, they can be located in different cellular compartments, including the nucleus, mitochondria, and cytoplasm, with the latter being the most frequent. This pattern is consistent with reports in *G. hirsutum* [34] *Z. mays* [40] and *Vitis* spp. [43], where some CPKs with post-translational modifications were detected in compartments other than the membrane. Furthermore, in *T. aestivum* [49], it has been observed that *TaCPK3* and *TaCPK15* are associated with the plasma membrane even in the absence of post-translational modifications. This indicates that, although post-translational modifications may favour anchoring to the membrane, this association is complex and may be influenced by other factors that allow them to perform diverse and specialised functions within the cell.

Analysis of the promoter regions (Figure 4) of *CqCPK* genes reveals a diversity of *cis*-regulatory elements associated with hormonal signalling pathways, environmental stimuli and developmental processes, which could suggest the possible functions and regulatory mechanisms of the genes, giving them the ability to adapt to adverse conditions. The presence of elements related to phytohormone pathways (ABA, GA, MeJA, SA, and AUX), as well as motifs sensitive to abiotic stress (MBS, ARE, TC-rich repeats, and LTR), indicates that these genes could be integrated into adaptive response networks against adverse conditions such as salinity, drought, or low temperatures. In particular, *CqCPK12*, *CqCPK17*, *CqCPK20* and *CqCPK32* showed a high density of motifs associated with regulation by ABA, MeJA and typical stress response elements such as ARE, MBS and TC-rich repeats. This combination suggests that these genes could be integrated into rapid and efficient signalling pathways against oxidative damage and osmotic imbalance induced by salt stress, reinforcing their functional role as sensors and modulators of the adaptive response. Similar findings have been reported in species such as *G. uralensis* [37], *G. hirsutum* [34], *C. capsularis* [38], *Setaria italica* [50], and *Carya illinoinensis* [29]. These studies have confirmed that these elements are common in genes involved in responses to abiotic stress, such as salinity, drought and cold.

Together, the *CqCPK* genes, especially *CqCPK12*, *CqCPK17*, *CqCPK20*, and *CqCPK32*, show structural and regulatory characteristics that predict their involvement in the response to abiotic stress. Their structural conservation and the presence of *cis*-elements associated with hormonal and environmental signals reinforce their potential role as key modulators in the adaptation of *C. quinoa* to adverse conditions.

### 3.2. Evaluation of Salt Tolerance in a Halotolerant and a Halosensitive Accession of C. quinoa

*C. quinoa* is recognised for its salinity tolerance. Studies conducted by Al-Naggar et al. and Prajapat et al. [50,51] have shown that this capacity varies significantly between genotypes and stages of development, reflecting a differential genotypic and phenological response to salt stress. In this study, accessions UNSA_VP021 and UNSA_VP033 showed contrasting responses to salt stress during germination.

The absolute decrease (DA) rate in germination (Figure 5A) showed a negative impact of the saline treatment compared to the control. The accessions showed a differential response to salinity stress, particularly above 500 mM, with accession UNSA_VP021 showing a reduction of 96 to 100%, being the most affected, indicating high sensitivity to saline stress. Meanwhile, accession UNSA_VP033 showed a germination DA of only 36%, suggesting greater tolerance. These results confirm that a higher DA is associated with greater susceptibility to salinity, as reported by Ravelombola et al. [52]. Similarly, the inhibition index (II), widely used as a parameter to assess the impact of stress on plants, followed the same pattern, with UNSA_VP021 showing a very high II (above 90%), reinforcing its profile in line with that reported by Qureshi et al. [53]. In contrast, the salt tolerance index (STI) reflects a plant’s ability to maintain its yield under saline conditions [54], highlighting that higher STI values indicate a greater probability of salt tolerance. In this study, significant differences were observed between accessions, with UNSA_VP033 presenting the highest STI (above 0.64). These results suggest that accession UNSA_VP021 is highly sensitive to salinity, while UNSA_VP033 exhibits marked tolerance during the germination stage.

In addition, a direct relationship was observed between tolerance indices and root length under saline conditions, with accession UNSA_VP033 maintaining a greater root length compared to UNSA_VP021, whose root development was severely affected by the increase in salinity. Although both accessions showed a significant reduction in root length as the NaCl concentration increased, UNSA_VP033 managed to sustain relatively superior root growth. These results are consistent with studies conducted on *A. thaliana* [55] and *Glycine max* [56], where salinity has been shown to reduce root growth, especially in sensitive genotypes.

Taken together, the results of the DA, II, and STI analyses and the root length evaluation clearly demonstrate that UNSA_VP021 is a sensitive accession, while UNSA_VP033 shows a tolerant response to salt stress, as described in the study by Alvarez-Vasquez et al. [57].

### 3.3. Analysis of Gene Expression in a Tolerant Accession and a Sensitive Accession in C. quinoa

The gene expression results for *CqCPK* genes reveal a differential response to salt stress in *C. quinoa*, with a clear contrast between accessions UNSA_VP033 (tolerant) and UNSA_VP021 (sensitive). In leaves (Figure 6), UNSA_VP033 showed significant overexpression of *CqCPK12*, *CqCPK17*, *CqCPK20*, and *CqCPK32* at 200 mM, a pattern that was maintained even at 300 mM (see Figure A1). This sustained activation suggests the involvement of these genes in the modulation of calcium-dependent mechanisms, possibly related to stomatal regulation, osmoprotectant production and redox balance (ROS) control. In contrast, in UNSA_VP021, all genes were found to be underexpressed at 200 mM, which would indicate a limited capacity for transcriptional activation in response to stress. The higher expression of *CqCPK* in the aerial part of UNSA_VP033 coincides with reports in other species such as *A. thaliana* [21,55], *O. sativa* [23,33], *G. hirsutum* [24], *A. comosus* [30], where *CPK* genes play key roles in signalling and adaptive response to salt stress.

In roots (Figure 7), expression at 200 mM was opposite; in the tolerant accession, all genes evaluated were underexpressed, except for *CqCPK17*, which remained stable. In contrast, in UNSA_VP021, expression was more variable. These results suggest that UNSA_VP033 focuses its adaptive response on the aerial part, probably thanks to more efficient ionic homeostasis, which includes Na^+^ exclusion in the root and regulated transport to the upper tissues. In contrast, the sensitive accession activates signalling pathways in the root, possibly as a strategy to limit Na^+^ absorption from the early stages of stress. This behaviour is supported by previous studies by Hariadi et al., Panuccio et al., and Claros et al. [58,59,60], which highlight the involvement of the root in ion exclusion and osmotic regulation under saline stress.

The variability in the expression of *CqCPK* genes between tissues and genotypes could represent a functional compensation strategy, where different organs assume specific roles to maintain homeostasis under stress. As pointed out by Kong et al. [61] and Zhang et al. [30] *CPK* gene expression can be highly tissue-specific, as in the case of *ZmCPK37* (root) and *ZmCPK22* (leaves) in maize, or *AcoCPK16* in pineapple leaves and flowers. Taken together, the differential expression of *CqCPKs* in roots and leaves suggests that *C. quinoa* accessions adopt divergent adaptive strategies in response to salinity. UNSA_VP033 appears to maintain a more efficient response from the aerial part, while UNSA_VP021 attempts to compensate through responses at the root level. Therefore, these results support the hypothesis that *CPK* genes are critical components in the signalling pathways that modulate the response to salt stress.

### 3.4. Analysis of Antioxidant Enzymes in a Tolerant Accession and a Sensitive Accession in C. quinoa

Salinity generates an excess of reactive oxygen species (ROS), compromising cell integrity and triggering oxidative damage. Plants counteract this effect through an antioxidant system that includes key enzymes such as superoxide dismutase (SOD) and peroxidase (POD) [60,62].

In this study, antioxidant activity was evaluated in *C. quinoa* leaves at 200 mM NaCl. The tolerant accession UNSA_VP033 showed significantly higher SOD and POD activity (*p* < 0.05) compared to the sensitive UNSA_VP021, suggesting a greater ability to neutralise ROS and protect cell structures from salt stress. This advantage was also reflected in lower malondialdehyde (MDA) accumulation in UNSA_VP033, indicating lower lipid peroxidation and better membrane integrity, which are essential for maintaining cell viability under salinity.

These results coincide with the overexpression of the genes *CqCPK12*, *CqCPK17*, *CqCPK20*, and *CqCPK32* in UNSA_VP033 leaves at 200 and 300 mM, suggesting that these calcium-dependent protein kinases could activate antioxidant pathways associated with tolerance. Previous research supports this role of *CPKs* in *OsCPK12* in *O. sativa* [23], *TaCPK27* in *T. aestivum* [26], and other CPKs in *A. thaliana* [21,55] and *Solanum habrochaites* [27], where these proteins have been shown to regulate ROS accumulation and actively participate in cellular defence mechanisms. Thus, our findings reinforce the hypothesis that *CqCPKs* play a key role in the activation of antioxidant mechanisms, contributing to greater tolerance to salt stress.

## 4. Materials and Methods

### 4.1. Identification of CPK family sequences in C. quinoa

To identify CPK genes in *C. quinoa*, the sequences of CPK genes from *A. thaliana* were downloaded from the database “The Arabidopsis Information Resource” (TAIR; http://www.arabidopsis.org). The CCDS of *A. thaliana* were used as input sequences to perform a sequence similarity search using the BLAST tool of the Phytozome v13 platform (https://phytozome-next.jgi.doe.gov/ (accessed on 17 March 2025)) and that of the National Centre for Biotechnology Information (NCBI; https://www.ncbi.nlm.nih.gov/ (accessed on 17 March 2025)) to obtain the genomic, coding, and protein sequences of *CPK* genes in *C. quinoa*.

The CqCPK amino acid sequences obtained were sent to InterProScan (https://www.ebi.ac.uk/interpro/search/sequence-search (accessed on 17 March 2025)), CDD v3.19 (https://www.ncbi.nlm.nih.gov/Structure/bwrpsb/bwrpsb.cgi (accessed on 17 March 2025)), and SMART v9.0 (http://smart.embl-heidelberg.de/ (accessed on 17 March 2025)) to verify the presence of the conserved serine/threonine kinase protein domain and EF-hand motifs. Redundant sequences were manually removed. Sequences with characteristic domains were assigned the code Cq (*C. quinoa*), followed by the letters CPK and an Arabic numeral.

### 4.2. Physicochemical Characterisation, Prediction of Characteristic Domains, Structure Analysis, and Prediction of Conserved Motifs

The characterisation of the physicochemical properties of CqCPKs, including gene sequence length, CDS length, amino acid sequence length, protein molecular weight, isoelectric point, and aliphatic index, was predicted using the ExPASy ProtParam web tool (https://web.expasy.org/protparam/ (accessed on 19 March 2025)). Post-translational modifications such as N-myristoylation and N-palmitoylation were performed using the Myristoylator server (http://web.expasy.org/myristoylator/ (accessed on 19 March 2025)) and the CSS-Palm v4.0 software programme, respectively. Subcellular localisation was predicted using the GENSCRIPT web tool (https://www.genscript.com/wolf-psort.html (accessed on 19 March 2025)) and CELLO (http://cello.life.nctu.edu.tw (accessed on 19 March 2025))

The presence and position of the serine/threonine kinase domains and EF-hand domains of CqCPKs were identified using the NCBI CDD v3.19 tool (https://www.ncbi.nlm.nih.gov/Structure/bwrpsb/bwrpsb.cgi (accessed on 19 March 2025)). The quinoa GFF annotation file was used for gene structure analysis. This file was downloaded from the quinoa database in Phytozome v13 (https://phytozome-next.jgi.doe.gov/ (accessed on 19 March 2025)). Multiple Em for Motif Elicitation (MEME) v5.1.1 (https://meme-suite.org/meme/tools/meme (accessed on 19 March 2025) software was used for the analysis of conserved motifs, and all graphs were visualised using TBtools v2.019 software.

### 4.3. Sequence Alignment and Phylogenetic Analysis

Multiple sequence alignment of *C. quinoa* proteins was performed using Clustal Omega software (https://www.ebi.ac.uk/jdispatcher/msa/clustalo (accessed on 20 March 2025)) with default parameters, and the results were visualised in JALVIEW v2.11.4.1 software. The aligned sequences were used to perform phylogenetic analysis using Molecular Evolutionary Genetic Analysis (MEGA v.11).

To construct the phylogenetic tree, the amino acid sequences of CPKs from *C. quinoa* and *A. thaliana* were used. The algorithm used was Neighbour-Joining with the Jones–Taylor–Thornton (JTT) substitution model, with gamma distribution (G), employing a run of 1000 repetitions and cut-off values of 50%. The image was visualised using the Newick file in the iTOL software v.7.2.2 (https://itol.embl.de/ (accessed on 20 March 2025)).

### 4.4. Analysis of the Promoter Regions of Elements Acting in cis on CqCPK Genes

To perform this analysis, the promoter sequence 2000 bp upstream of the start codon of the CqCPKs was used. These regions were then analysed in the PlantCARE database (https://bioinformatics.psb.ugent.be/webtools/plantcare/html/ (accessed on 20 March 2025)) to predict the elements acting in *cis*. These results were visualised using TBtools v2.019 software.

### 4.5. Plant Material and Treatments Performed

Two accessions characterised by Alvarez et al. [57] were used, from the Germplasm Bank of the Laboratory of Genetic Resources and Molecular Genetics of the National University of San Agustin de Arequipa, one of them as halotolerant (UNSA_VP033) and the other as halosensitive (UNSA_VP021).

a. Evaluation during the germination phase

The procedure was carried out according to Alanoca et al. [63] With modifications, where the quinoa seeds were disinfected with 2% sodium hypochlorite for 5 min and then rinsed repeatedly with distilled water. They were then placed in Petri dishes on filter paper with 5 mL of NaCl at concentrations of 100 mM, 200 mM, 300 mM, 400 mM, 500 mM, 600 mM and 700 mM, with distilled water as a control group. Three repetitions of 100 seeds each were performed. They were then incubated in a germination chamber at a temperature of 22 °C, with a photoperiod of 11 h/13 h light/darkness, the light intensity was 3233.3 ± 11.6 lux, measured using a digital light meter (Model GM1010, Benetech, Palo Alto, CA, USA). Seed germination was evaluated after 5 days of exposure to the treatment. Seeds were considered germinated when the radicle length exceeded 0.2 mm. The following formula was used to calculate the germination percentage [64]:

$$Germination\;percentage=\frac{\left(Number\;of\;germinated\;seeds\right)}{\left(Total\;number\;of\;seeds\;analyzed\right)}\times 100\%$$

The effect of salt stress on quinoa seed germination was evaluated by calculating the absolute decrease index (AD), inhibition index (II), and salt tolerance index (ITS), which were calculated based on the following equations [52]:

$$absolute\;decrease\;index\left(AD\right)=GC-GT\;$$

$$inhibition\;index\left(II\right)=\frac{GC-GT}{GC}*\;100\;$$

$$salt\;tolerance\;index(ITS)=\frac{GT*\;GC}{{{GC}_{av}}^{2}}\;$$

where GC = number of seeds germinated in the control group, GT = number of seeds germinated under salt stress, and GC_av_ = average germination of the control group.

b. Evaluation of radicle growth

To evaluate radicle growth, the seeds were disinfected by immersing them in 0.5% CuSO_4_ for 5 min and rinsed five times with sterile distilled water. They were then treated with 2% NaOCl and 300 µL of Tween 80 for 15 min and rinsed five times with sterile distilled water. Ten seeds were then sown in a Petri dish with 1× Murashige-Skoog (MS) medium (Table A2) supplemented with different concentrations of NaCl (100 mM, 200 mM, 300 mM, 400 mM). They were placed vertically in a growth chamber and incubated with a 16/8 hours’ light/dark photoperiod at a temperature of 25 °C. Radicle growth was evaluated 7 days after sowing, determining their length in millimetres by manual measurement with a graduated millimetre scale sheet.

c. Obtaining quinoa seedlings subjected to salt stress under hydroponic conditions.

A salt-tolerant accession (UNSA_VP033) and a salt-sensitive accession (UNSA_VP021) were used. The seeds were first disinfected with 2% sodium hypochlorite, followed by 10 rinses with distilled water, and then germinated on moist filter paper for 4 days at a temperature of 21 °C. Following the protocol described by Cole et al. [65], modified, the seedlings were transferred to hydroponic containers containing Hoagland’s nutrient solution at a concentration of 1x (Table A2). The system was placed in a growth chamber at a temperature of 24 °C, with a photoperiod of 16/8 h of light and darkness, and a humidity of 50–70%. After 24 days, when the plants had developed their third true leaf, they were exposed to salinity treatments with sodium chloride. Knowing the median lethal dose of each accession reported by Alvarez et al. [57], three salinity treatments were applied to the tolerant accession: 100 mM, 200 mM, and 300 mM; and two treatments were applied to the sensitive accession: 100 mM and 200 mM. In both cases, control treatments were included, in which no salinity treatment was applied. Each treatment consisted of a container with 28 seedlings. The treatments were carried out in triplicate. After 5 days of treatment, the leaves and roots were collected, placed in liquid nitrogen, and stored at 80 °C.

d. Primer Design

Primers were designed based on the obtained CqCPK sequences (Table 2). These primers were designed to have a melting temperature (Tm) between 58 °C and 61 °C, a length of 18 to 25 base pairs, and an amplicon length of 100 to 200 bp, using Primer3Plus (https://www.bioinformatics.nl/cgi-bin/primer3plus/primer3plus.cgi (accessed on 25 March 2025)).

### 4.6. RNA Extraction and Gene Expression Analysis by qPCR

100 mg of roots and 90 mg of leaves were ground in liquid nitrogen to obtain a fine powder. For total RNA extraction, the specifications of the Spectrum Plant Total RNA kit manufacturer (Sigma-Aldrich-Merck, Darmstadt, Germany) were followed. Concentration and purity were measured in a spectrophotometer at 260 and 280 nm (Biotech Epock 2, Winooski, VT, USA), and the RNA extracts were stored at −80 °C until use.

For cDNA synthesis, 5 µg of RNA was used, and following the specifications of the GoTaq^®^ 2-Step RT-qPCR Kit (Promega, Madison, WI, USA) manufacturer, the product obtained was stored at −20 °C until use.

The GoTaq^®^ 2-Step RT-qPCR kit (Promega, Madison, WI, USA) was used for the qPCR protocol. The mixture consisted of 1 µL of forward primers, 1 µL of reverse primers, 4 µL of cDNA, 4 µL of nuclease-free water, and 10 µL of SYBR green master mix. The thermal cycling conditions of the thermocycler were: 95 °C for 2 min of preheating, followed by 40 cycles of 95 °C for 15 s of denaturation and 61 °C for 1 min of annealing and elongation.

For gene expression analysis, three biological samples per treatment were processed with three technical replicates for each qPCR run. The relative expression levels of the *CqCPK* genes were calculated using the 2(−∆∆CT) method. The reference gene Glyceraldehyde-3-phosphate dehydrogenase b (GAPDH, GenBank ID: XM_021862063.1) was used for internal control.

### 4.7. Determination of Lipid Peroxidation

**Figure A2:**
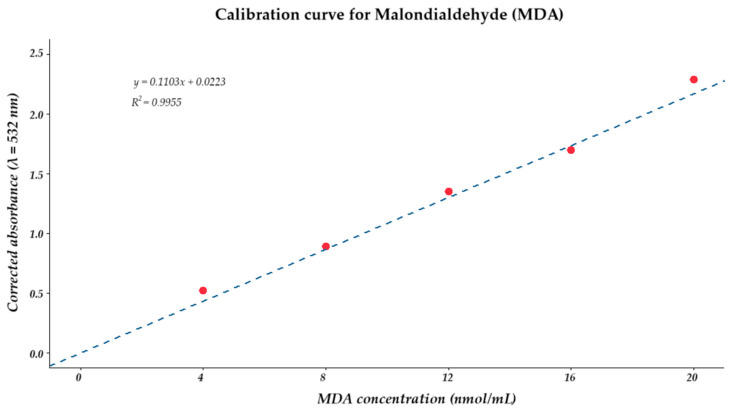
Calibration curve for the quantification of malondialdehyde (MDA). Standard MDA solutions were analysed in a range of 4 to 20 µM, in triplicate. Red dots represent experimental absorbance values, and the dotted line indicates the linear regression fitted to the data. The absorbance values were corrected by subtracting the blank and plotted against concentration.

Membrane lipid peroxidation was determined by estimating the malondialdehyde (MDA) content in leaves. Ten milligrams of *C. quinoa* leaves were weighed, ground to a fine powder in liquid nitrogen, and then 300 µL of lysis buffer with 3 µL BTH (butylhydroxytoluene) was added. After centrifugation at 13,000× *g* for 10 min, the supernatant was used to determine the MDA content. The amount of malondialdehyde was measured using the lipid Peroxidation (MDA) Assay Kit, MAK568 (Sigma-Aldrich, Merck KGaA, Darmstadt, Germany), which is based on the detection and quantification of the product formed by the reaction of MDA and thiobarbituric acid (TBA) under acidic conditions. The resulting product was detected spectrophotometrically at 532 nm. For quantification, a calibration curve (Figure A2) was constructed using known MDA standards, and the results were expressed in nano moles per millilitre (nmol/mL).

### 4.8. Determination of SOD and POD Enzyme Activity

To detect enzyme activities, 0.05 g of leaf were ground in liquid nitrogen to obtain a fine powder, then suspended in 300 µL of buffer (0.1 M Tris-HCl, pH 7.4; 0.5% Triton X-100 and 5 mM β-mercaptoethanol), the mixture was centrifuged at 14,000× *g* for 5 min, and the resulting supernatant was used to determine the enzymatic activity of superoxide dismutase (SOD) and peroxidase (POD). Commercial kits were used for both determinations.

**Figure A3:**
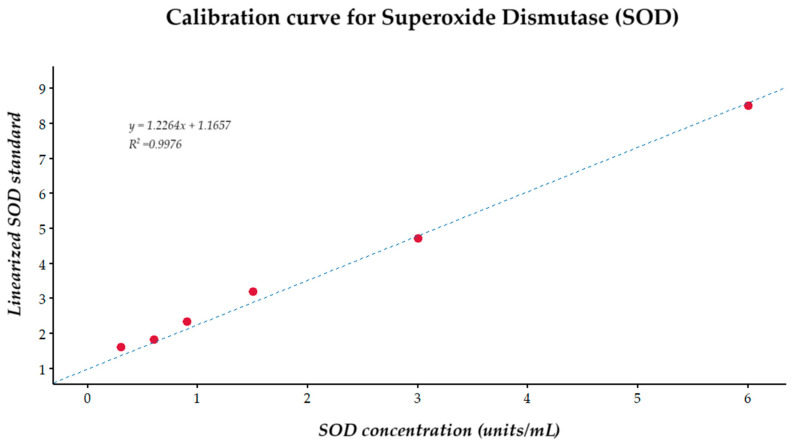
Calibration curve for the quantification of superoxide dismutase (SOD) activity. Red dots represent experimental absorbance values, and the dotted line indicates the linear regression fitted to the data. The absorbance values were corrected by subtracting the blank and then linearized using the ratio between the corrected absorbance of the control without SOD (NO SOD) and each sample.

SOD activity was measured using the SOD Assay Kit, SOD-CC009BUL (Sigma-Aldrich, Merck KGaA, Darmstadt, Germany) based on the inhibition of the reduction of tetrazolium salt by superoxide anions generated by the xanthine/xanthine oxidase system. For quantification, a calibration curve (Figure A3) was constructed using different known concentrations of SOD, and enzyme activity was calculated from the inhibition of the formation of the coloured compound, whose pigment was measured in an Epoch 2 microplate spectrophotometer (Biotech Epock 2, Winooski, VT, USA). The results were expressed in units per millilitre of protein (U/mL).

Peroxidase (POD) activity was measured using the Peroxidase Assay Kit, MAK506 (Sigma-Aldrich, Merck KGaA, Darmstadt, Germany)which is based on a colorimetric reaction in which the enzyme catalyses the oxidation of a chromogenic substrate in the presence of hydrogen peroxide (H_2_O_2_). The substrate included in the kit produces a resorufin product upon oxidation (λ = 570 nm), and the intensity of the coloured product is directly proportional to enzyme activity. POD activity was expressed as micromoles of product formed per minute per volume of protein (µM/min).

For the interpretation of the results, the values obtained in each treatment were corrected by calculating the difference with respect to its corresponding control (treatment-control). This correction allowed the net effect of salt stress on enzyme activity to be evaluated exclusively, minimising the variability associated with baseline conditions between accessions.

### 4.9. Statistical Analysis

Statistical analysis was performed to evaluate differences in salt tolerance indices during germination, relative gene expression, lipid peroxidation, and enzyme activity in two accessions, one sensitive and one tolerant. To compare the means between the two accessions, Student’s *t*-test for independent samples was applied, under the assumptions of normality and homogeneity of variances, with a significance level set at *p* < 0.05. To analyse rootlet growth under salinity conditions and to evaluate the differential behaviour of CPK genes in the tolerant accession compared to 100, 200, and 300 mM NaCl relative to the control, a one-way ANOVA was used, considering salinity concentration as a factor. When the ANOVA indicated significant differences (*p* < 0.05), Tukey’s post hoc test (HSD) was applied to perform multiple comparisons between treatments. All analyses were performed in R Studio version 4.4.2.

Additional tables and figures supporting this study are provided in the Supplementary Materials.

## 5. Conclusions

The present study identified and characterised 16 members of the calcium-dependent protein kinase family in *C. quinoa*, demonstrating that they play a fundamental role in the adaptive response to salt stress, with the genes CqCPK12, CqCPK17, CqCPK20, and CqCPK32 standing out, which show significantly higher expression in the tolerant accession UNSA_VP033 under saline stress conditions. These results suggest that these genes play a key role in regulating ion homeostasis and antioxidant responses, contributing to salt tolerance.

These findings provide a solid basis for functional studies aimed at improving quinoa varieties to find more resilient varieties, as well as for their application in programmes aimed at improving crop adaptation to extreme climatic environments, contributing to the development of more sustainable agriculture and strengthening global food security.

## Figures and Tables

**Table 1:** Physicochemical properties of the proteins encoded by the CPK genes in *Chenopodium quinoa*.

Gene Name	Gene Report	Localization	Gene (pb)	CDS (pb)	PP (aa)	PM (kDa)	pI	IA	N-Myr	N-Pal.	N-terminal	Cellular Location
*CqCPK1*	AUR62014281	Scaffold_1566:453457..459507 r	6050	1797	599	66.440	5.08	80.07	No	Yes	MGNTCVRP	Cy ^1^ Mt ^2^
*CqCPK3*	AUR62019775	Scaffold_1480:652411..664554 r	12143	1551	516	58.896	6.06	84.67	No	No	MAYPPLSL	Cy ^1^ Mt ^2^
*CqCPK4A*	AUR62013181	Scaffold_1040:2957000..2963439 f	6439	1707	568	63.716	5.36	82.22	No	Yes	MGNTCRGS	Cy ^1,2^
*CqCPK4B*	AUR62004642	Scaffold_4250:5773748..5778723 f	4975	1614	537	59.373	5.22	84.45	No	No	MSENKQGG	Cy ^1^ Nu ^2^
*CqCPK7*	AUR62019309	Scaffold_3107:1905680..1910192 f	4512	1590	529	59.249	6.49	83.71	No	Yes	MGNCCGSP	Cy ^1^ Mt ^2^
*CqCPK8*	AUR62000129	Scaffold_2088:1440132..1445975 f	5843	1596	531	59.505	6.49	84.48	No	Yes	MGNCCGSP	Cy ^1^ Mt ^2^
*CqCPK9*	AUR62015780	Scaffold_1529:2804144..2808576 f	4432	1599	532	59.608	5.87	78.12	Yes	Yes	MGICASKN	Cy ^1^ Nu ^2^
*CqCPK10*	AUR62021752	Scaffold_1675:3323192..3327703 r	4511	1623	540	61.281	6.47	86.09	Yes	Yes	MGNCNACI	Cy ^1^ Mt ^2^
*CqCPK12*	AUR62000624	Scaffold_2088:6685549..6689015 f	3466	1482	493	55.695	4,94	84.24	No	No	MTFNKTNS	Cy ^1,2^
*CqCPK16*	AUR62003366	Scaffold_1000:23399212..23404508 r	5296	1788	595	66.797	9.15	81.29	Yes	Yes	MGSCLSSS	Cy ^1^ Nu ^2^
*CqCPK17*	AUR62006551	Scaffold_3429:2828367..2830818 r	2451	1602	533	59.944	5.98	76.85	No	Yes	MGNCCPRG	Cy ^1^ Mt ^2^
*CqCPK20*	AUR62038418	Scaffold_2310:558276..566238 f	7962	1413	470	52.599	5.02	92.49	No	No	MGLQLESV	Cy ^1^ Nu ^2^
*CqCPK24*	AUR62015413	Scaffold_2751:6423932..6426773 r	2841	1626	541	61.623	6.49	82.33	Yes	Yes	MGGCISTF	Cy ^1^ Go ^2^
*CqCPK28*	AUR62029467	Scaffold_3036:826063..831054 f	4991	1575	524	59.446	9	82.81	No	No	MVAKTAAK	Cy ^1^ Nu ^2^
*CqCPK29*	AUR62013888	Scaffold_1412:3722500..3726911 r	4411	1431	476	54.345	5.53	88.7	No	No	MSSIPEHD	Cy ^1^ Nu ^2^
*CqCPK32*	AUR62026022	Scaffold_3792:1752559..1756451 f	3892	1590	529	60.098	6.47	86.82	No	Yes	MGNCCIRP	Cy ^1^ Mt ^2^

Abbreviations: (r) reverse, (f) forward N-Myr: N-myristoylation sites, N-Pal: palmitoilation sites CDS: bp: base pairs, CDS sequence; PP: protein size, aa: amino acids; kDa: kilodalton; pI: isoelectric point; Cy: Cytoplasm; Nu: Nucleus; Mt: Mitochondria; Go: Golgi. ^1^ Subcellular localisation was predicted using CELLO v.2.5 (http://cello.life.nctu.edu.tw/ (accessed on 19 March 2025)), ^2^ Subcellular localisation was predicted using GenScript (https://www.genscript.com/psort.html (accessed on 19 March 2025)). Key sites of post-translational modification: Glycine (G) at position 2 for N-myristoylation and Cysteines (C) for S-palmitoylation.

**Table 2:** Primers used in CqCPK sequences.

Code	Gen		Primers
**AUR62019775**	CqCPK3	>CqCPK3_F	TGGCTTACCCTCCACTCTCT
		>CqCPK3_R	AGAATAATTGCTGCGACGGTTG
**AUR62013181**	CqCPK4A	>CqCPK4A_F	TCAAGAACAGGGCCTTAACGA
		>CqCPK4A_R	ACGCATGGTTCTCCTTCCAA
**AUR62004642**	CqCPK4B	>CqCPK4B_F	GAAAGTTGGGCTGAAGAAAGTG
		>CqCPK4B_R	TCACATGAAGGGTAGCAGCAA
**AUR62015780**	CqCPK9	>CqCPK9A_F	ACAAGCTCAAGCAACTAGCG
		>CqCPK9A_R	CGGGCCAATCCCTCTTTCAA
**AUR62021752**	CqCPK10	>CqCPK10_F	AGGAGAAATTATGGGCCAGAGG
		>CqCPK10_R	GCACTCCTCGTAAGATAGCCA
**AUR62000624**	CqCPK12	>CqCPK12_F	GATGAACTCCAGCAAGCCT
		>CqCPK12_R	TCCATTGCCCATCCTCATCA
**AUR62003366**	CqCPK16	>CqCPK16_F	TCCAAATCTTGACGCTGCTAGG
		>CqCPK16_R	GTGATGACACCAATGCTCCAA
**AUR62006551**	CqCPK17	>CqCPK17_F	AAAGGGTGTTACCTCCACCTC
		>CqCPK17_R	CCCGACTTGGATAGGCTTGG
**AUR62038418**	CqCPK20	>CqCPK20_F	GACAACAGCGGCACAATAGA
		>CqCPK20_R	ACATCTCCCAAACCGAACTG
**AUR62029467**	CqCPK28	>CqCPK28_F	GATGATACTTCCAATCGCTGTG
		>CqCPK28_R	TCTCCACCTTCACATAACTCCA
**AUR62013888**	CqCPK29	>CqCPK29A_F	*TTTGGGCTCTCTGTCTTCATCG*
		>CqCPK29A_R	AAATGACTCCAGCACTCCACA
**AUR62026022**	CqCPK32	>CqCPK32_F	TGTGTGAAGGAGGCGAGTTG
		>CqCPK32_R	TTCTCGGGTTTCAGGTCACG

## Data Availability

The data presented in this study are available upon request from the corresponding author.

## Supplementary Materials

The following supporting information can be downloaded at: https://www.mdpi.com/article/10.3390/ijms262110658/s1.

## Appendix A. Additional Information

**Table A1:** Motif, sequence, and function of the *cis*-elements presented in Figure 2.

Cis-Element	Sequence	Function
**Plant Hormone**		
**ABRE**	ACGTG	*cis*-acting element involved in the abscisic acid responsiveness
**TGACG-motif**	TGACG	*cis*-acting regulatory element involved in the MeJA-responsiveness
**CGTCA-motif**	CGTCA	*cis*-acting regulatory element involved in the MeJA-responsiveness
**TATC-box**	TATCCCA	*cis*-acting element involved in gibberellin-responsiveness
**TCA-element**	CCATCTTTTT	*cis*-acting element involved in salicylic acid responsiveness
**P-box**	CCTTTTG	gibberellin-responsive element
**GARE-motif**	TCTGTTG	gibberellin-responsive element
**TGA-element**	AACGAC	auxin-responsive element
**AuxRR-core**	GGTCCAT	*cis*-acting regulatory element involved in auxin responsiveness
**Stress Responsiveness**		
**GC-motif**	CCCCCG	enhancer-like element involved in anoxic-specific inducibility
**LTR**	CCGAAA	*cis*-acting element involved in low-temperature responsiveness
**MBS**	CAACTG	MYB binding site involved in drought-inducibility
**TC-rich repeats**	GTTTTCTTAC	*cis*-acting element involved in defence and stress responsiveness
**WUN-motif**	AAATTTCCT	wound-responsive element
**Plant Growth**		
**ARE**	AAACCA	*cis*-acting regulatory element essential for the anaerobic induction
**CAT-box**	GCCACT	*cis*-acting regulatory element related to meristem expression
**circadian**	CAAAGATATC	*cis*-acting regulatory element involved in circadian control
**GCN4_motif**	TGAGTCA	*cis*-regulatory element involved in endosperm expression
**MBSI**	AAAAAAC(G/C)GTTA	MYB binding site, involved in flavonoid biosynthetic genes regulation
**O2-site**	GATGATGTGG	*cis*-acting regulatory element involved in zein metabolism regulation
**RY-element**	CATGCATG	*cis*-acting regulatory element involved in seed-specific regulation
**Light Responsiveness**		
**G-Box**	CACGTT	*cis*-acting regulatory element involved in light responsiveness
**AAAC-motif**	CAATCAAAACCT	light-responsive element
**GT1-motif**	GGTTAAT	light-responsive element
**Sp1**	GGGCGG	light-responsive element
**MRE**	AACCTAA	MYB binding site involved in light responsiveness
**ATC-motif**	AGTAATCT	part of a conserved DNA module involved in light responsiveness
**ATCT-motif**	AATCTAATCC	part of a conserved DNA module involved in light responsiveness
**Box 4**	ATTAAT	part of a conserved DNA module involved in light responsiveness
**chs-CMA1a**	TTACTTAA	part of a light-responsive element
**GA-motif**	ATAGATAA	part of a light-responsive element
**Gap-box**	CAAATGAA(A/G)A	part of a light-responsive element
**GATA-motif**	AAGATAAGATT	part of a light-responsive element
**I-box**	AGATAAGG	part of a light-responsive element
**TCCC-motif**	TCTCCCT	part of a light-responsive element
**TCT-motif**	TCTTAC	part of a light-responsive element
**AE-box**	AGAAACAA	part of a module for light response
**chs-Unit 1 m1**	ACCTAACCCGG	Light-responsive element
**3-AF1 binding site**	TAAGAGAGGAA	Light-responsive element
**ACE**	GACACGTATG	*cis*-acting element involved in light responsiveness
**AT1-motif**	AATTATTTTTTATT	part of a light-responsive module
**Box II**	CCACGTGGC	part of a light-responsive element
**LAMP-element**	CTTTATCA	part of a light-responsive element
**CAG-motif**	GAAAGGCAGAC	Light-responsive element
**GATT-motif**	CTCCTGATTGGA	part of a light-responsive element

**Table A2:** Composition of MS medium [66], supplemented with different concentrations of sodium chloride (NaCl).

Component	Concentration (mg/L)
Macronutrients	
Ammonium nitrate (NH_4_NO_3_)	1650
Potassium nitrate (KNO_3_)	1900
Monopolyphosphate (KH_2_PO_4_)	170
Magnesium sulphate (MgSO_4_·7H_2_O)	370
Calcium chloride (CaCl_2_·2H_2_O)	440
Micronutrients	
Boric acid (H_3_BO_3_)	6.2
Manganese sulphate (MnSO_4_·H_2_O)	22.3
Zinc sulphate (ZnSO_4_·7H_2_O)	8.6
Potassium iodide (KI)	0.83
Sodium molybdate (Na_2_MoO_4_·2H_2_O)	0.25
Copper sulphate (CuSO_4_·5H_2_O)	0.025
Cobalt chloride (CoCl_2_·6H_2_O)	0.025
Iron (Fe-EDTA)	
Ferrous sulphate (FeSO_4_·7H_2_O)	27.8
Disodium EDTA (Na_2_EDTA)	37.3
Carbon source	
Sucrose	30,000 (30 g)
Agar	7500 (7.5 g)
pH adjustment	5.7 ± 0.1
Sterilisation	Autoclave 121 °C, 20 min
Treatments	
concentration	NaCl (g/L)
Control	0
100 mM	5.84
200 mM	11.69
300 mM	17.53
400 mM	23.38

**Table A3:** Mineral nutrient solutions according to Hoagland.

Symbol	Component	Concentration	
		Molar	g/L
**A**	Ca(NO_3_)_2_.4H_2_O	1.00 M	236.00
**B**	KNO_3_	1.00 M	101.00
**C**	MgSO_4_.7H_2_O	1.00 M	247.00
**D**	KH_2_PO_4_	1.00 M	136.00
**E**	Micronutrients		
**F**	Fe-EDTA		
**Micronutrients**	Concentration g/L	Micronutrients	Concentration g/L
**MnCl_2_.4H_2_O**	1.81	H_3_BO_3_	2.86
**ZnSO_4_.7H_2_O**	0.22	CuSO_4_.5H_2_O	0.10
**H_2_MoO_4_.H_2_O**	0.10		

## Appendix B

**Table A4:** Germination response to different salinity concentrations in *C. quinoa* accessions.

Accession	Repetition	Concentration							
		0 mM	100 mM	200 mM	300 mM	400 mM	500 mM	600 mM	700 mM
UNSA_VP021	1	100	95	93	39	22	5	0	0
	2	100	96	95	41	24	3	0	0
	3	100	94	94	40	23	4	0	0
UNSA_VP033	1	100	100	99	99	95	93	78	64
	2	100	100	99	98	94	94	76	65
	3	100	100	99	97	96	95	77	66

**Table A5:** Salinity tolerance indices in the germination phase of the UNSA_VP033 and UNSA_VP021 accessions of *C. quinoa* under different NaCl concentrations.

Accesion	INDEX	Repetition	Concentration						
			100 mM	200 mM	300 mM	400 mM	500 mM	600 mM	700 mM
UNSA_VP021	Absolute decrease	1	5	7	61	78	95	100	100
		2	4	5	59	76	97	100	100
		3	6	6	60	77	96	100	100
	Inhibition index	1	5	7	61	78	95	100	100
		2	4	5	59	76	97	100	100
		3	6	6	60	77	96	100	100
	Salt tolerance index	1	0.95	0.93	0.39	0.22	0.05	0	0
		2	0.96	0.95	0.41	0.24	0.03	0	0
		3	0.94	0.94	0.4	0.23	0.04	0	0
UNSA_VP033	Absolute decrease	1	0	1	1	5	7	22	36
		2	0	1	2	6	6	24	35
		3	0	1	3	4	5	23	34
	Inhibition index	1	0	1	1	5	7	22	36
		2	0	1	2	6	6	24	35
		3	0	1	3	4	5	23	34
	Salt tolerance index	1	1	0.99	0.99	0.95	0.93	0.78	0.64
		2	1	0.99	0.98	0.94	0.94	0.76	0.65
		3	1	0.99	0.97	0.96	0.95	0.77	0.66

**Table A6:** Response to Salinity at Different Concentrations in a Tolerant Accession and a Sensitive Accession of *C. quinoa* in Root Length (mm).

Accession	Repetition	0 mM	100 mM	200 mM	300 mM	400 mM
UNSA_VP021	1	17	10	7	2	0
	2	16	9	6	3	0
	3	18	11	5	4	0
UNSA_VP033	1	68	61	49	12	7
	2	70	62	48	10	8
	3	71	60	50	11	6

**Table A7:** Response to Salinity at Different Concentrations in a tolerant accession and a sensitive accession of *C. quinoa* in the contents MDA, SOD, POD.

Accession		Repetition	Treatment	
			CONTROL	200 mM
UNSA_VP021	MDA	1	10.402	22.188
		2	11.067	22.007
		3	10.644	22.430
	SOD	1	88.677	71.865
		2	90.047	70.811
		3	88.002	72.398
	POD	1	18.444	12.111
		2	17.178	11.778
		3	17.778	11.644
UNSA_VP033	MDA	1	3.330	5.869
		2	3.451	5.929
		3	3.512	5.748
	SOD	1	54.812	85.361
		2	55.617	86.669
		3	54.413	84.715
	POD	1	14.111	18.444
		2	14.444	19.111
		3	14.911	18.711

**Table A8:** Response to Salinity at Different Concentrations in a sensitive accession (UNSA_VP021) of *C. quinoa* to analyse the expression levels of the CqCPK genes (%).

Organ	Treatment	Repetition	CqCPK3	CqCPK4A	CqCPK4B	CqCPK9	CqCPK10	CqCPK12	CqCPK16	CqCPK17	CqCPK20	CqCPK28	CqCPK29	CqCPK32
Root	0 mM	1	1.00	1.00	1.00	1.00	1.00	1.00	1.00	1.00	1.00	1.00	1.00	1.00
		2	1.00	1.00	1.00	1.00	1.00	1.00	1.00	1.00	1.00	1.00	1.00	1.00
		3	1.00	1.00	1.00	1.00	1.00	1.00	1.00	1.00	1.00	1.00	1.00	1.00
	100 mM	1	0.46	1.30	1.03	0.88	0.88	0.53	0.88	1.03	0.86	0.63	1.09	1.01
		2	0.46	1.28	0.99	0.84	0.84	0.56	0.90	1.00	0.87	0.61	1.04	1.03
		3	0.44	1.23	1.01	0.85	0.88	0.54	0.89	0.96	0.85	0.63	1.06	1.01
	200 mM	1	0.83	1.91	0.98	1.72	1.47	1.04	1.20	0.99	1.02	2.05	1.04	1.02
		2	0.85	1.95	1.03	1.64	1.42	1.00	1.23	0.97	1.01	1.99	1.04	0.99
		3	0.80	1.98	1.04	1.70	1.48	0.98	1.22	1.00	1.06	2.01	1.02	1.05
Leaf	0 mM	1	1.00	1.00	1.00	1.00	1.00	1.00	1.00	1.00	1.00	1.00	1.00	1.00
		2	1.00	1.00	1.00	1.00	1.00	1.00	1.00	1.00	1.00	1.00	1.00	1.00
		3	1.00	1.00	1.00	1.00	1.00	1.00	1.00	1.00	1.00	1.00	1.00	1.00
	100 mM	1	0.21	0.43	2.01	0.59	0.14	0.36	0.16	2.01	0.44	0.09	0.44	0.03
		2	0.22	0.43	2.14	0.58	0.13	0.37	0.16	2.12	0.41	0.09	0.44	0.03
		3	0.21	0.42	2.09	0.59	0.13	0.37	0.15	2.05	0.43	0.09	0.43	0.03
	200 mM	1	0.47	0.46	0.27	0.70	0.26	0.53	0.14	0.49	0.53	0.14	0.11	0.02
		2	0.46	0.44	0.25	0.72	0.25	0.53	0.14	0.48	0.54	0.13	0.10	0.02
		3	0.46	0.45	0.25	0.72	0.26	0.52	0.14	0.51	0.56	0.13	0.11	0.01

**Table A9:** Response to Salinity at Different Concentrations in a tolerant accession (UNSA_VP033) of *C. quinoa* to analyse the expression levels of the CqCPK genes (%).

Organ	Treatment	Repetition	CqCPK3	CqCPK4A	CqCPK4B	CqCPK9	CqCPK10	CqCPK12	CqCPK16	CqCPK17	CqCPK20	CqCPK28	CqCPK29	CqCPK32
Root	0 mM	1	1.00	1.00	1.00	1.00	1.00	1.00	1.00	1.00	1.00	1.00	1.00	1.00
		2	1.00	1.00	1.00	1.00	1.00	1.00	1.00	1.00	1.00	1.00	1.00	1.00
		3	1.00	1.00	1.00	1.00	1.00	1.00	1.00	1.00	1.00	1.00	1.00	1.00
	100 mM	1	0.92	0.45	0.48	0.66	0.51	0.79	0.51	1.00	0.91	0.25	0.46	1.01
		2	1.04	0.42	0.51	0.68	0.50	0.77	0.50	1.02	0.91	0.25	0.50	1.00
		3	1.02	0.45	0.50	0.69	0.50	0.76	0.49	0.97	0.93	0.26	0.50	1.03
	200 mM	1	0.61	0.30	0.24	0.32	0.33	0.73	0.21	0.96	0.38	0.07	0.29	0.53
		2	0.60	0.28	0.26	0.33	0.35	0.73	0.21	0.98	0.39	0.07	0.29	0.52
		3	0.63	0.30	0.25	0.33	0.33	0.71	0.20	0.98	0.38	0.07	0.28	0.53
	300 mM	1	1.06	0.25	0.24	0.20	0.32	0.41	0.47	0.91	0.39	0.16	0.52	0.27
		2	1.06	0.25	0.25	0.20	0.31	0.41	0.47	0.97	0.40	0.15	0.52	0.26
		3	1.09	0.26	0.25	0.20	0.30	0.40	0.45	0.93	0.40	0.15	0.53	0.25
Leaf	0 mM	1	1.00	1.00	1.00	1.00	1.00	1.00	1.00	1.00	1.00	1.00	1.00	1.00
		2	1.00	1.00	1.00	1.00	1.00	1.00	1.00	1.00	1.00	1.00	1.00	1.00
		3	1.00	1.00	1.00	1.00	1.00	1.00	1.00	1.00	1.00	1.00	1.00	1.00
	100 mM	1	0.93	0.33	0.32	0.20	0.23	0.68	1.10	0.94	0.99	1.57	0.26	1.67
		2	0.95	0.34	0.32	0.19	0.23	0.69	1.09	0.95	0.98	1.50	0.25	1.71
		3	0.94	0.32	0.30	0.20	0.23	0.66	1.13	0.98	0.97	1.49	0.25	1.70
	200 mM	1	1.20	0.74	0.53	0.60	0.41	1.84	0.51	1.85	2.03	1.08	0.48	2.08
		2	1.24	0.74	0.51	0.62	0.41	1.82	0.52	1.90	2.04	1.08	0.48	2.13
		3	1.27	0.71	0.50	0.63	0.42	1.83	0.52	1.90	2.02	1.04	0.49	2.07
	300 mM	1	0.82	0.22	0.95	0.23	0.12	1.15	1.15	1.80	2.08	0.62	0.52	1.99
		2	0.82	0.22	1.00	0.22	0.13	1.14	1.13	1.87	2.02	0.63	0.50	2.08
		3	0.78	0.22	0.97	0.22	0.13	1.17	1.17	1.89	2.04	0.64	0.50	2.04
