# Supplementary material for: In Silico Characterisation and Determination of Gene Expression Levels of the CPK Family Under Saline Stress Conditions in Chenopodium quinoa Willd
Source: Int J Mol Sci. 2025 Nov 1;26(21):10658. doi: 10.3390/ijms262110658 (PMC12609707; doi:10.3390/ijms262110658)
Supplement: Supplementary file 1 [file ijms-26-10658-s001.zip › ijms-3950414-supplementary/meltingcurves.pdf]

UNSA_VP033 – Roots - 0mM

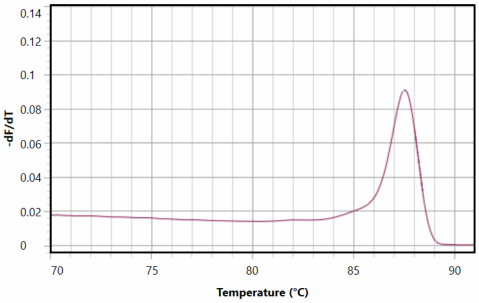

CqCPK3

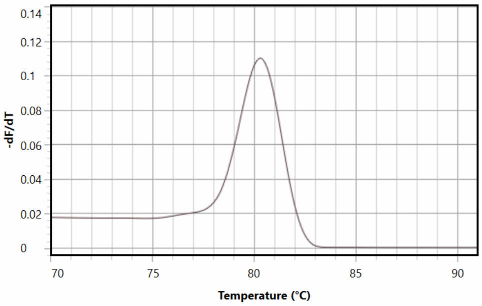

CqCPK4A

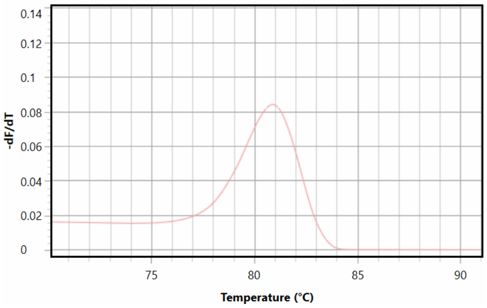

CqCPK4B

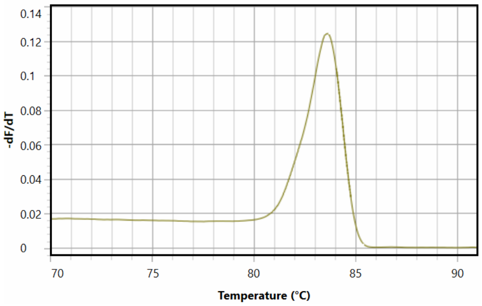

CqCPK9

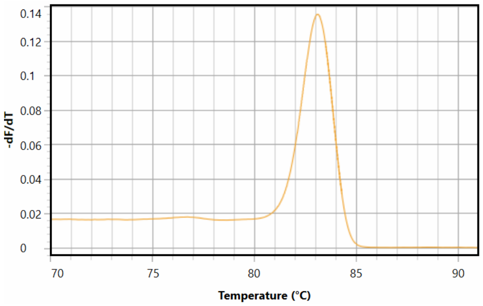

CqCPK10

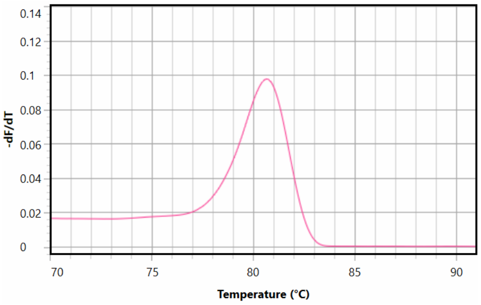

CqCPK12

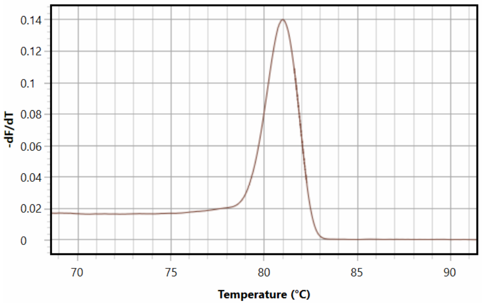

CqCPK16

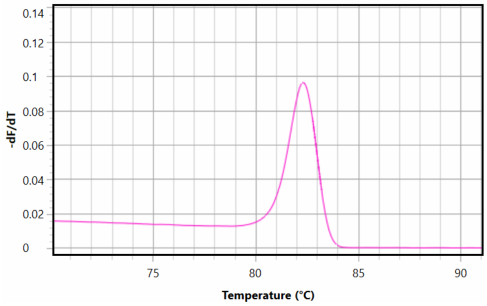

CqCPK17

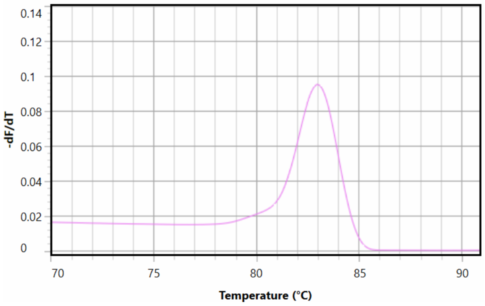

CqCPK20

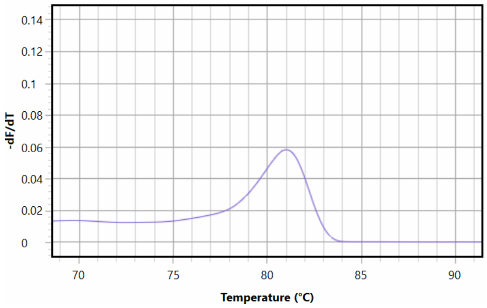

CqCPK28

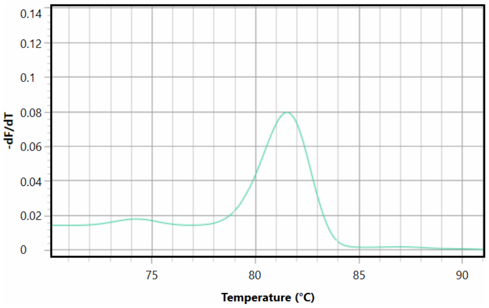

CqCPK29

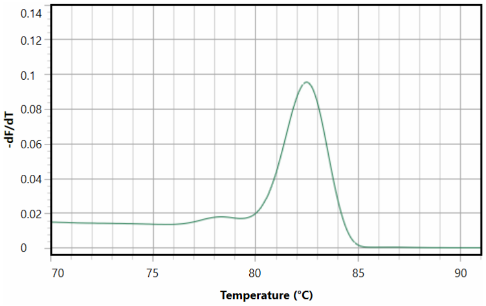

CqCPK32

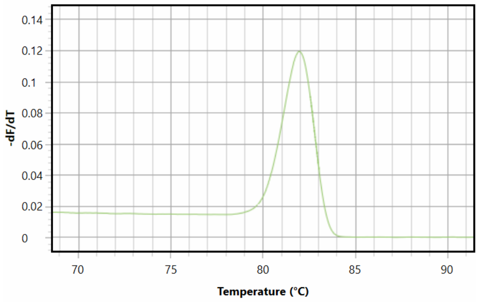

GAPDH-B

UNSA_VP033 - Roots – 100mM

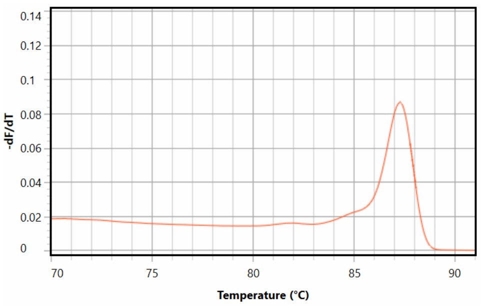

CqCPK3

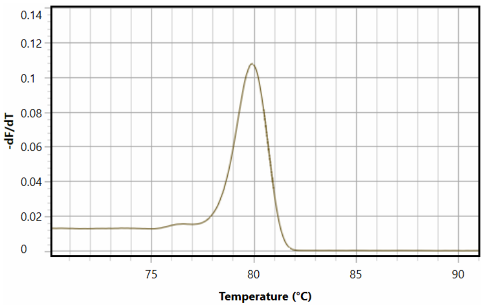

CqCPK4A

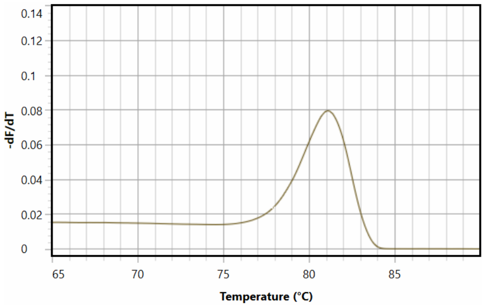

CqCPK4B

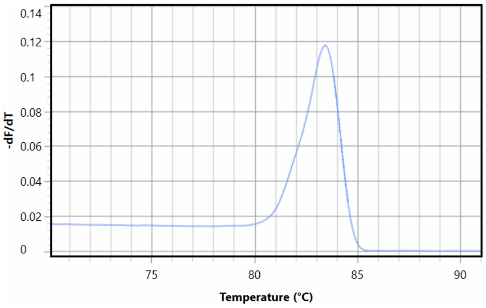

CqCPK9

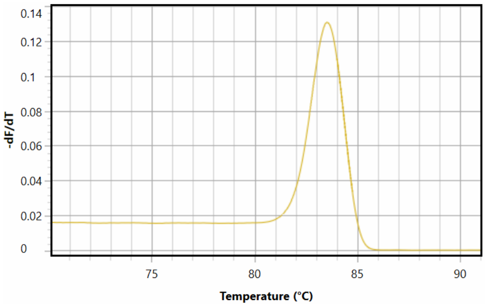

CqCPK10

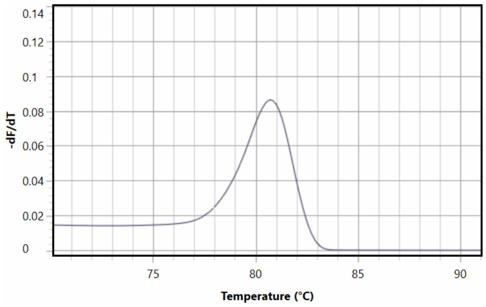

CqCPK12

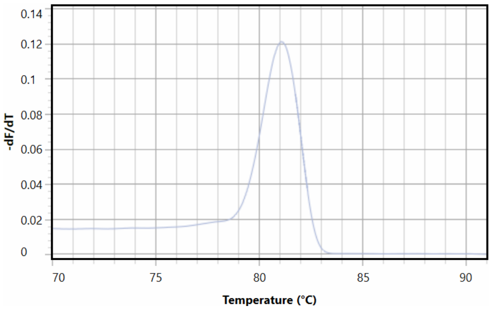

CqCPK16

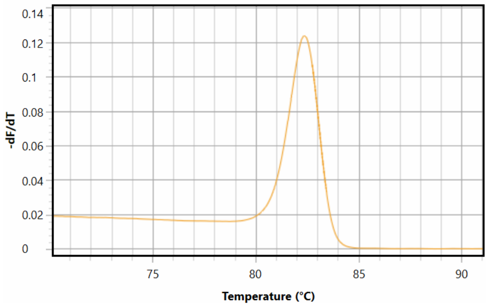

CqCPK17

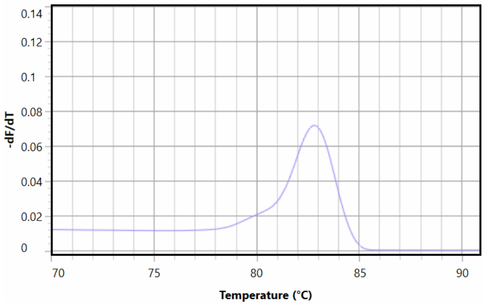

CqCPK20

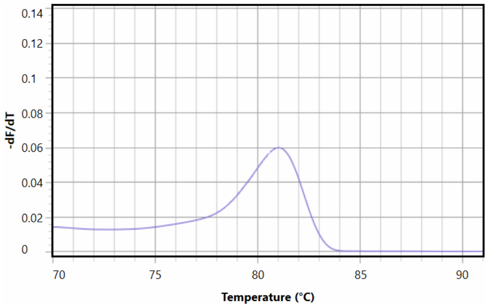

CqCPK28

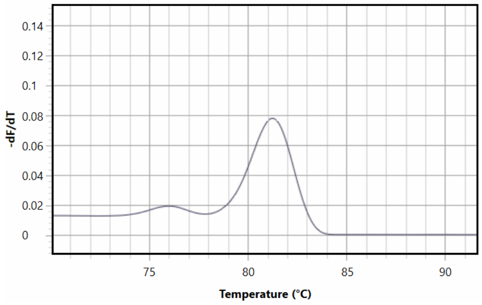

CqCPK29

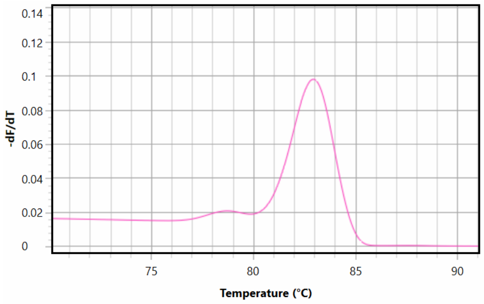

CqCPK32

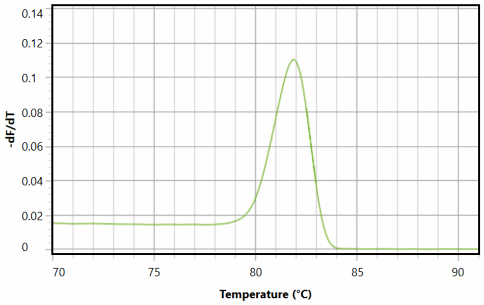

GAPDH-B

UNSA_VP033 - Roots – 200mM

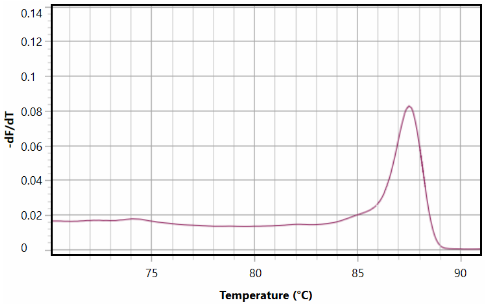

CqCPK3

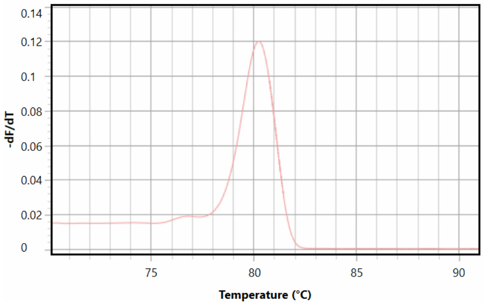

CqCPK4A

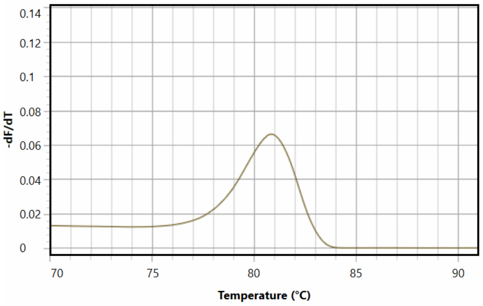

CqCPK4B

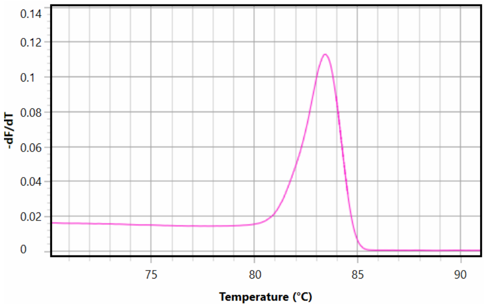

CqCPK9

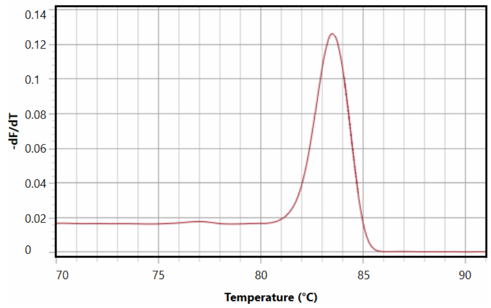

CqCPK10

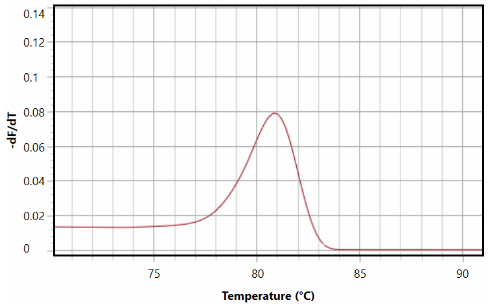

CqCPK12

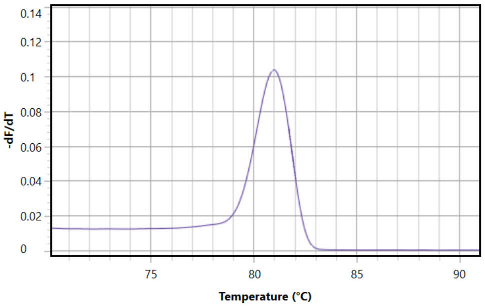

CqCPK16

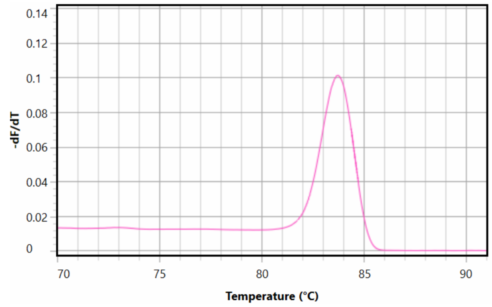

CqCPK17

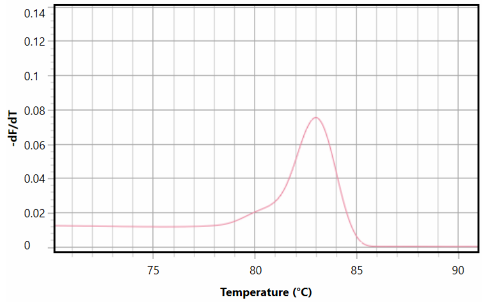

CqCPK20

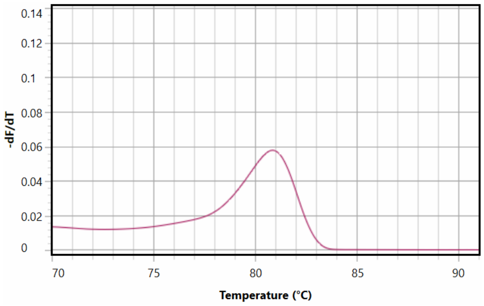

CqCPK28

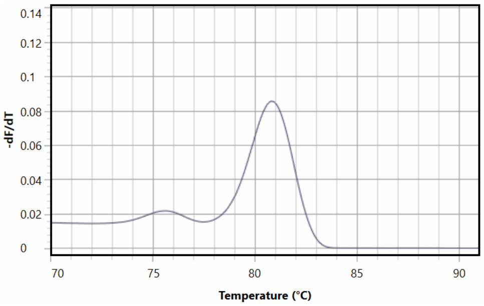

CqCPK29

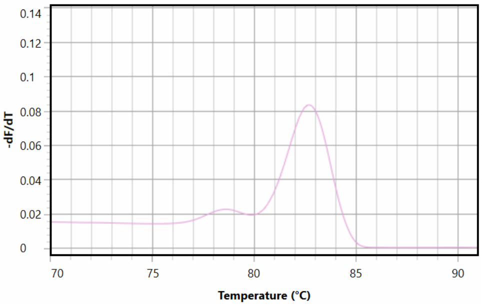

CqCPK32

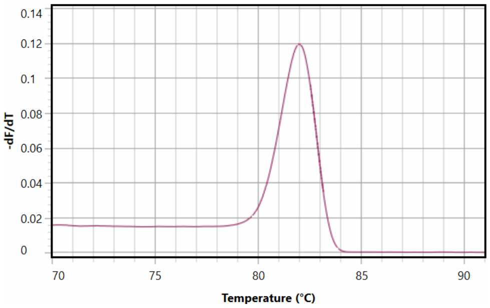

GAPDH-B

UNSA VP033 - Roots - 300 mM

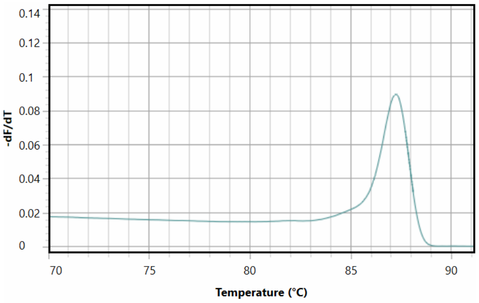

CqCPK3

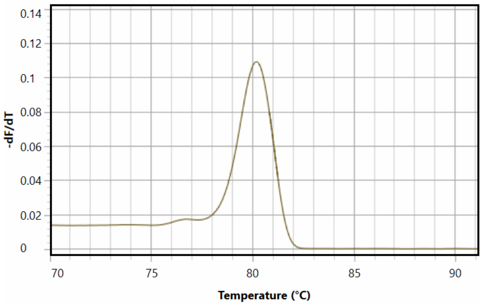

CqCPK4A

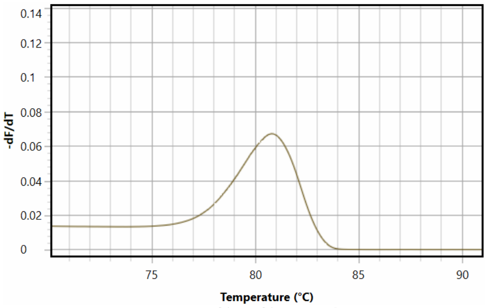

CqCPK4B

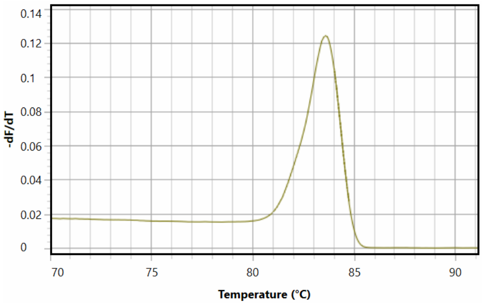

CqCPK9

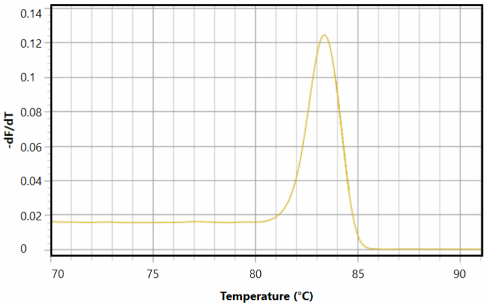

CqCPK10

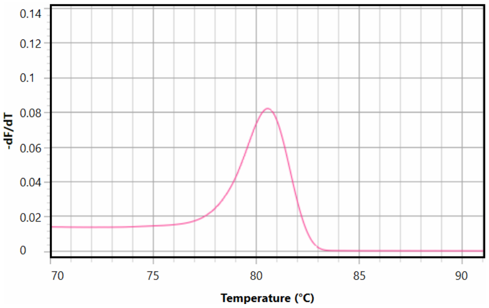

CqCPK12

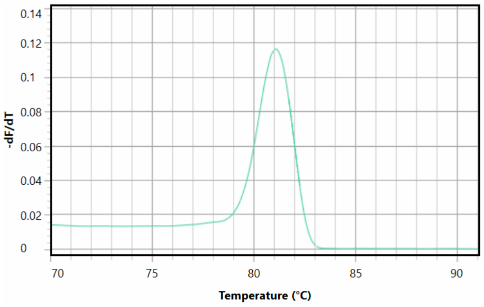

CqCPK16

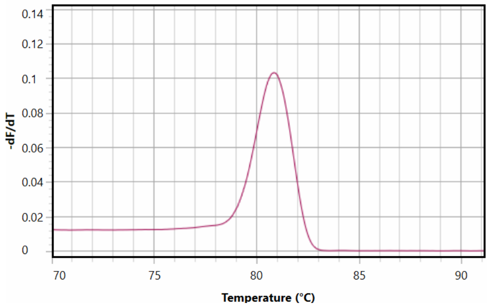

CqCPK17

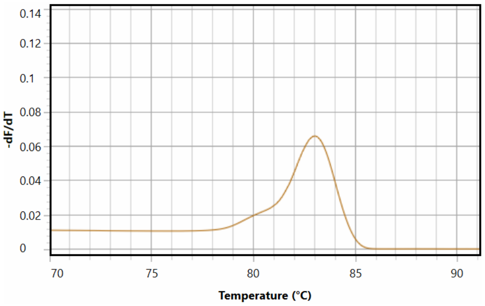

CqCPK20

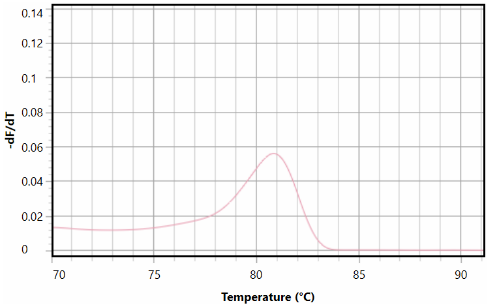

CqCPK28

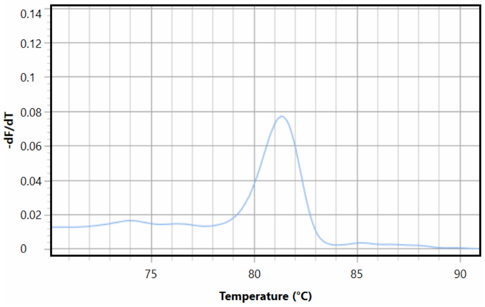

CqCPK29

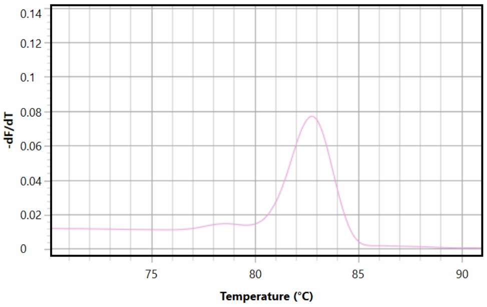

CqCPK32

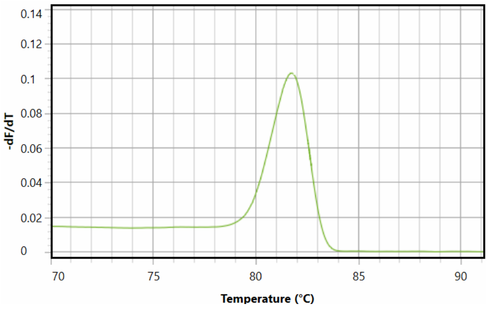

GAPDH-B

UNSA_VP021 - Roots - 0mM

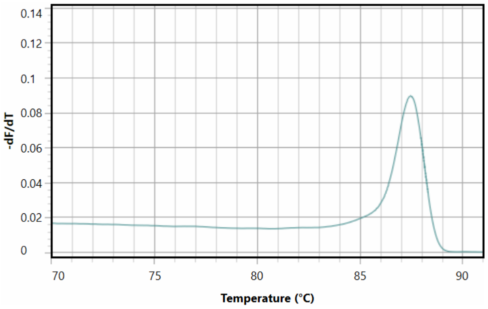

CqCPK3

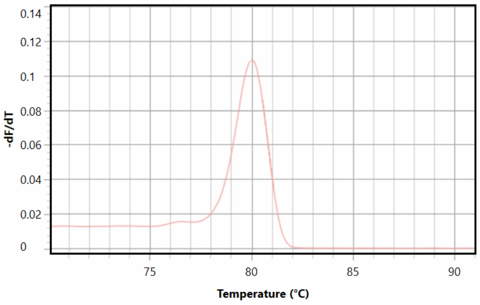

CqCPK4A

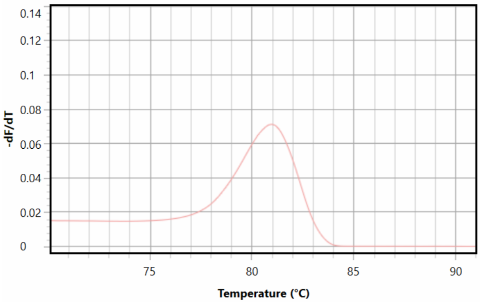

CqCPK4B

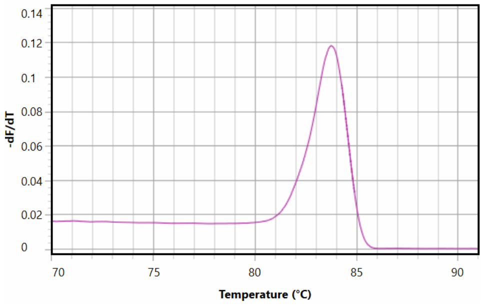

CqCPK9

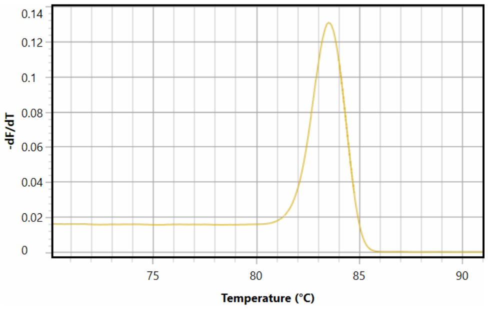

CqCPK10

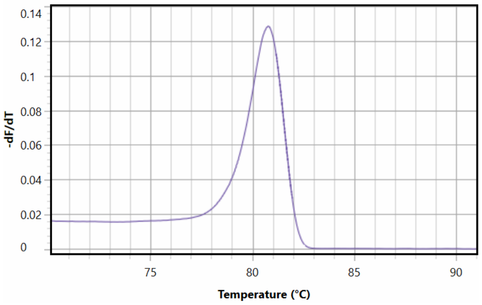

CqCPK12

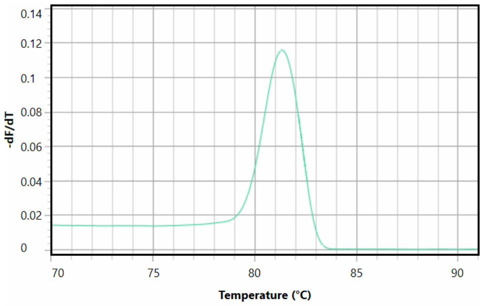

CqCPK16

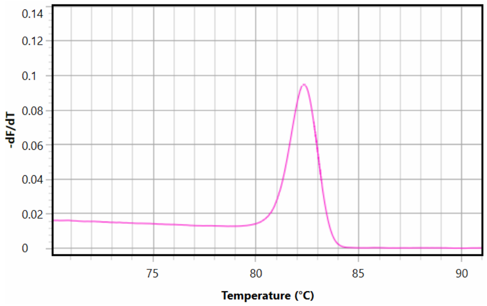

CqCPK17

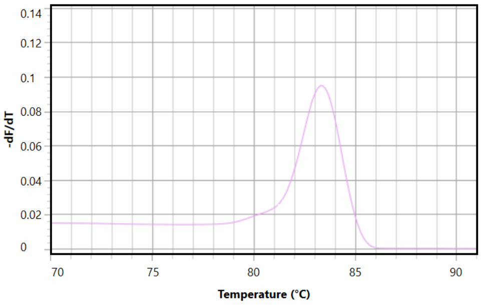

CqCPK20

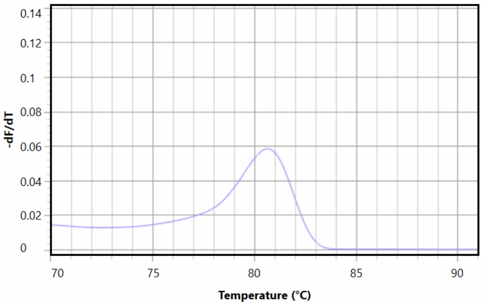

CqCPK28

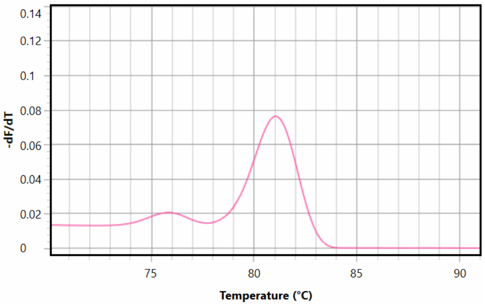

CqCPK29

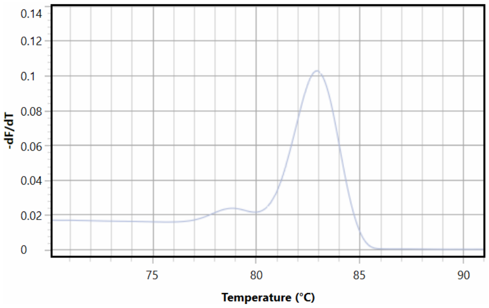

CqCPK32

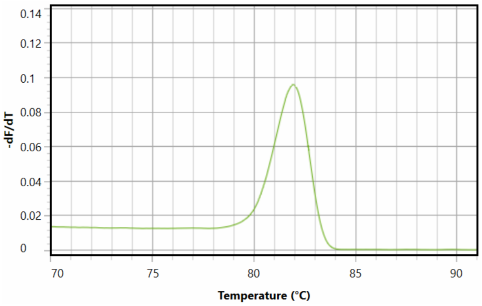

GAPDH-B

UNSA_VP021 - Roots - 100mM

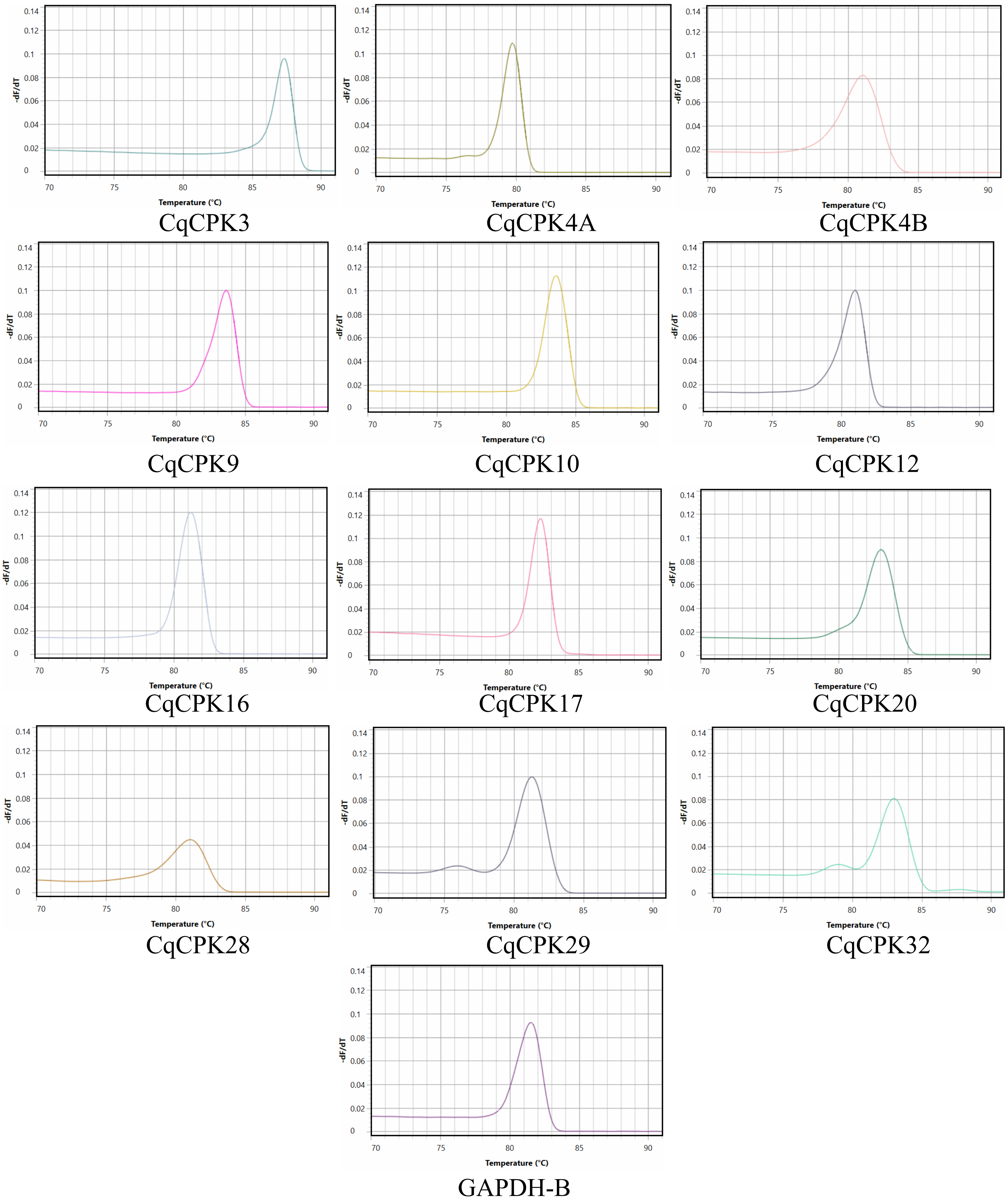

UNSA_VP021 - Roots - 200mM

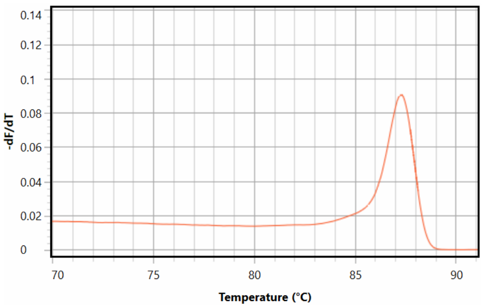

CqCPK3

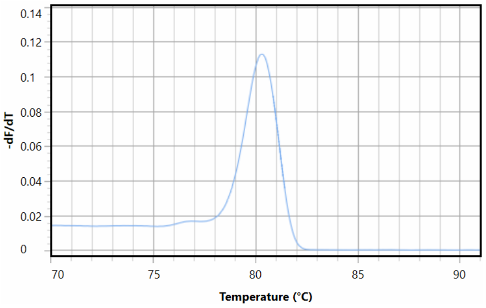

CqCPK4A

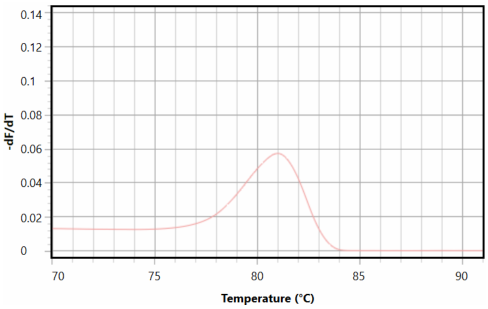

CqCPK4B

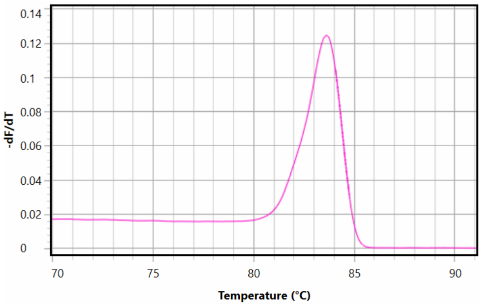

CqCPK9

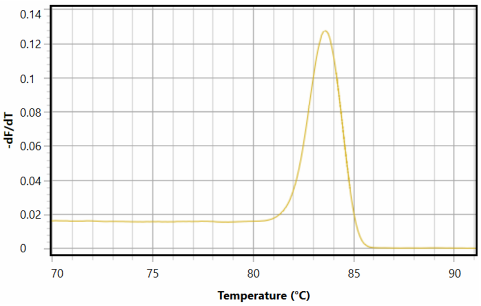

CqCPK10

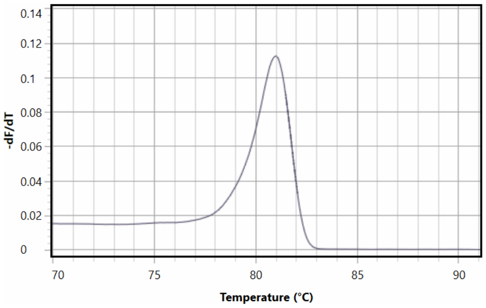

CqCPK12

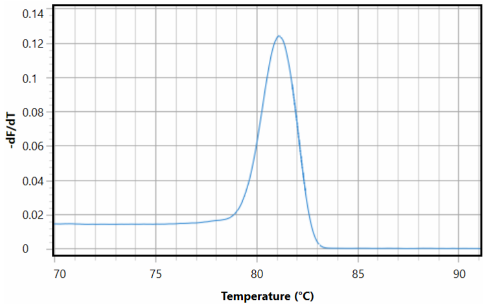

CqCPK16

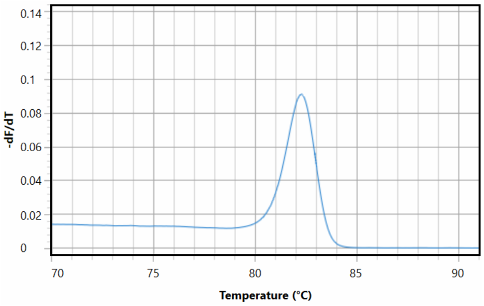

CqCPK17

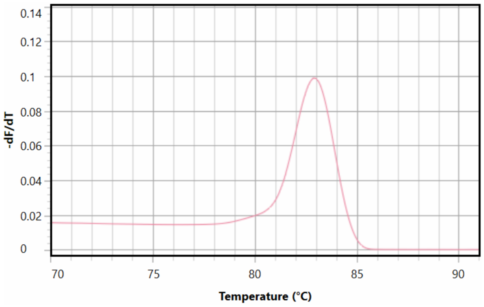

CqCPK20

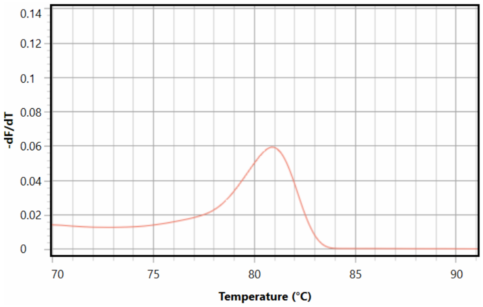

CqCPK28

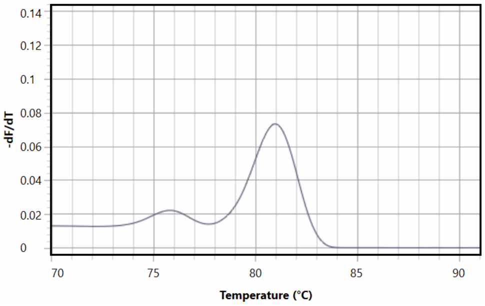

CqCPK29

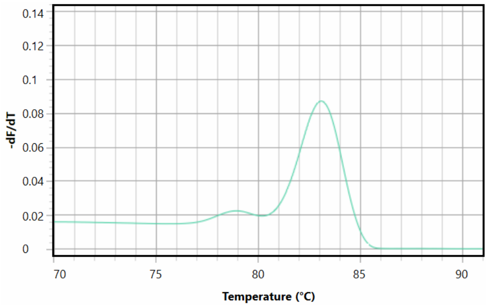

CqCPK32

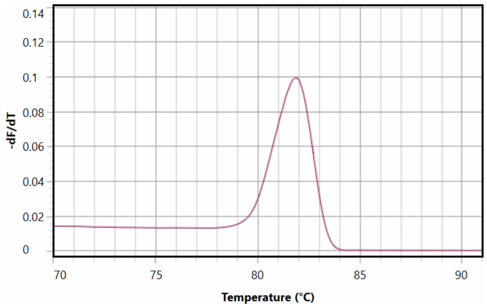

GAPDH-B

UNSA_VP033 - Leaves – 0mM

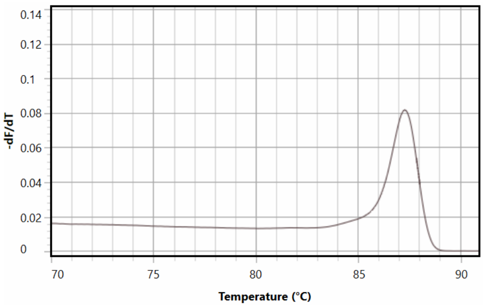

CqCPK3

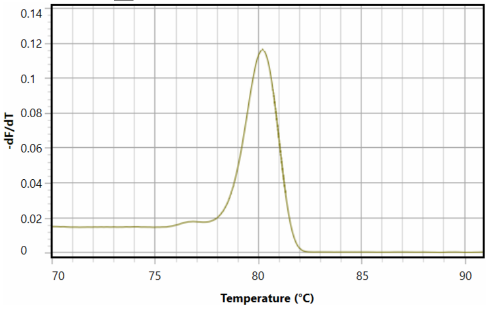

CqCPK4A

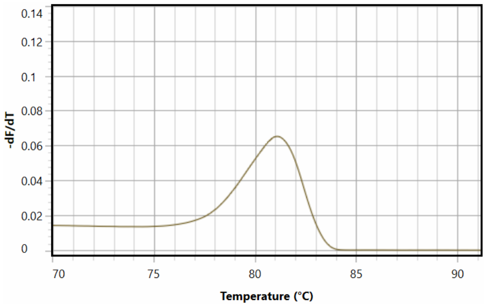

CqCPK4B

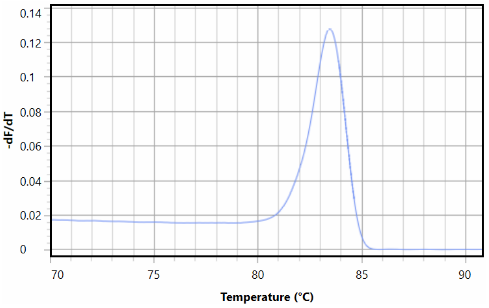

CqCPK9

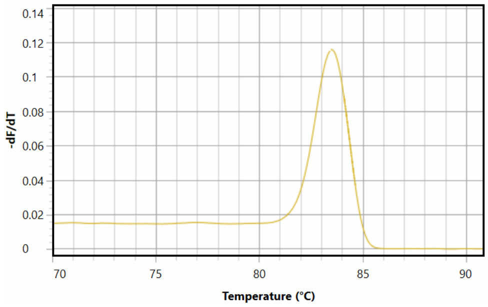

CqCPK10

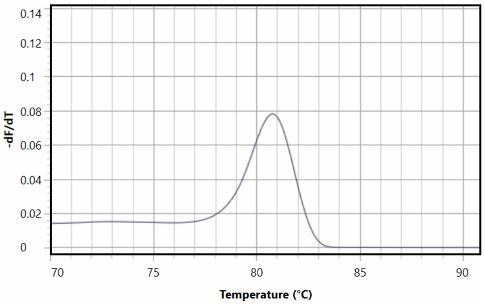

CqCPK12

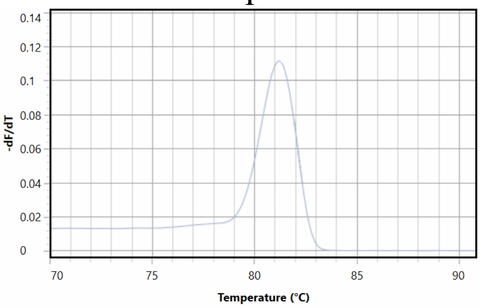

CqCPK16

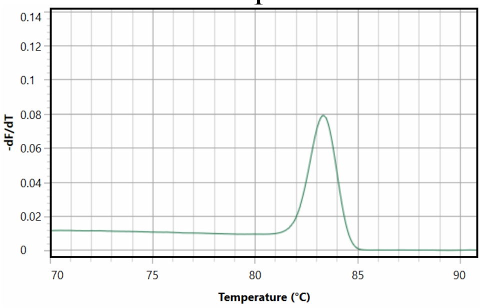

CqCPK17

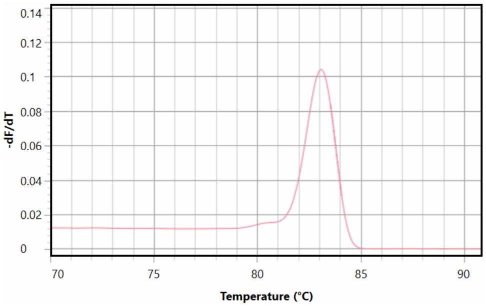

CqCPK20

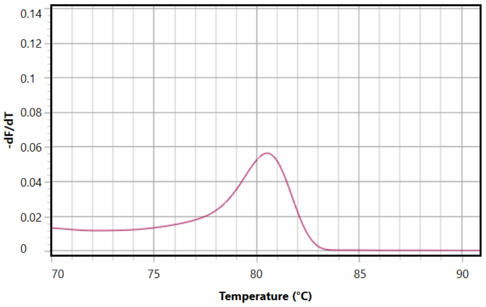

CqCPK28

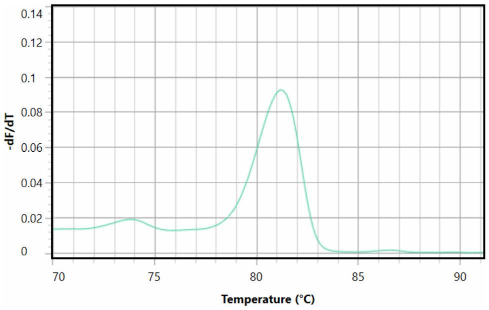

CqCPK29

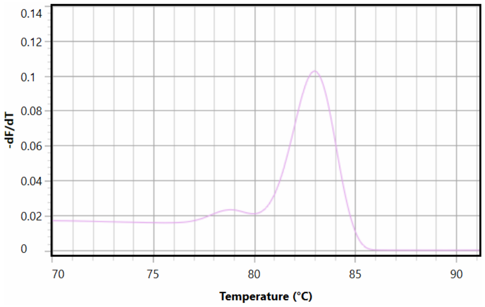

CqCPK32

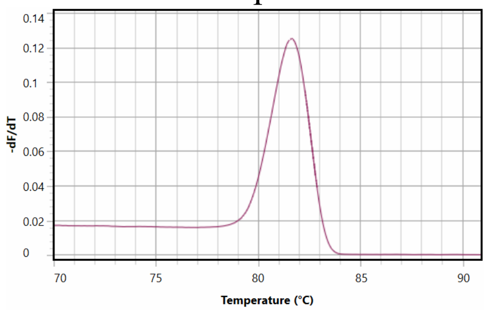

GAPDH-B

UNSA_VP033 - Leaves - 100mM

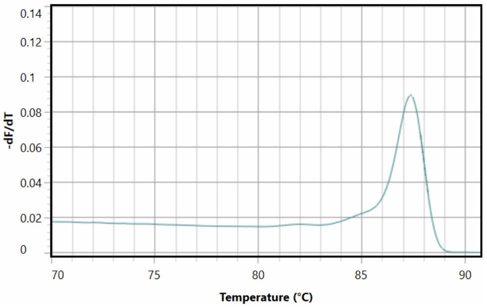

CqCPK3

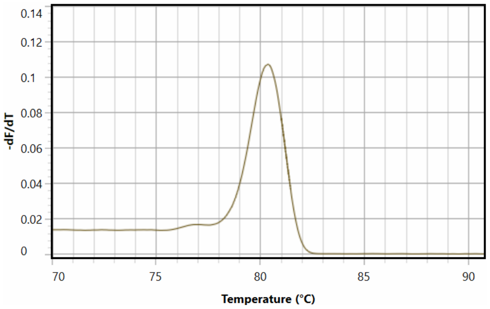

CqCPK4A

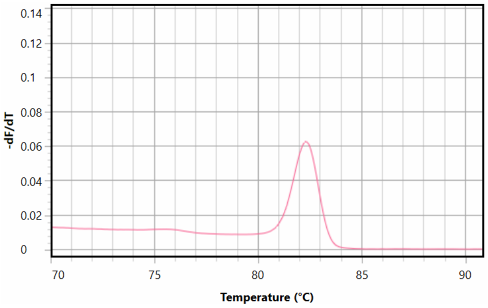

CqCPK4B

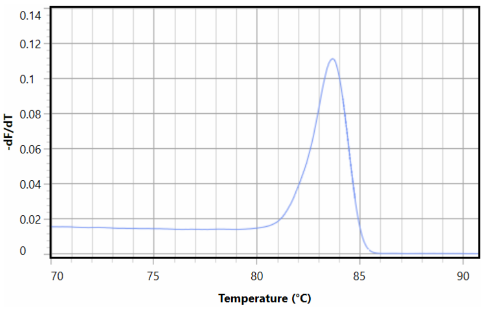

CqCPK9

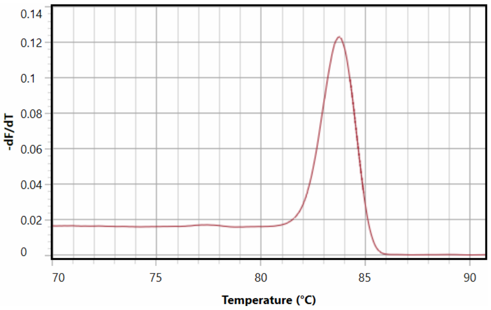

CqCPK10

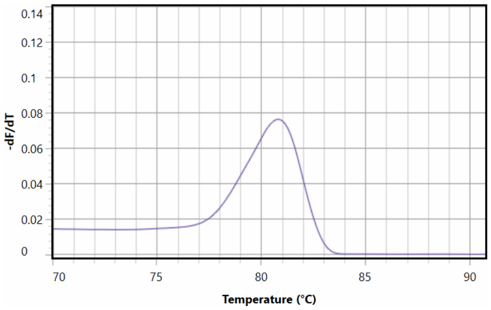

CqCPK12

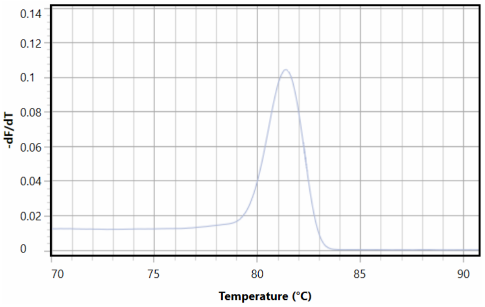

CqCPK16

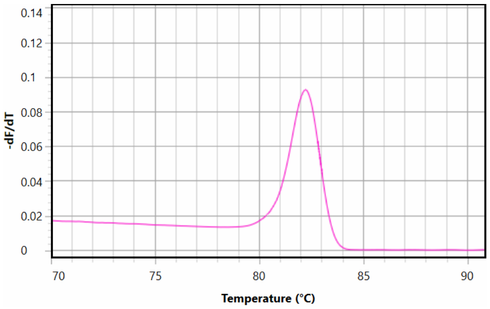

CqCPK17

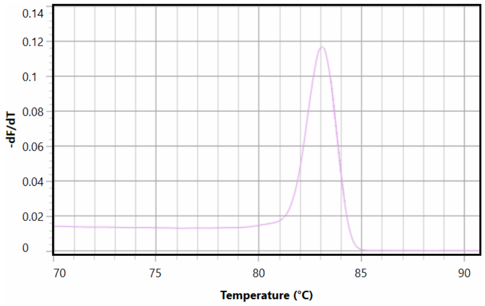

CqCPK20

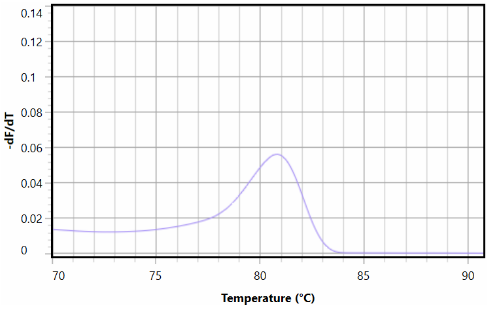

CqCPK28

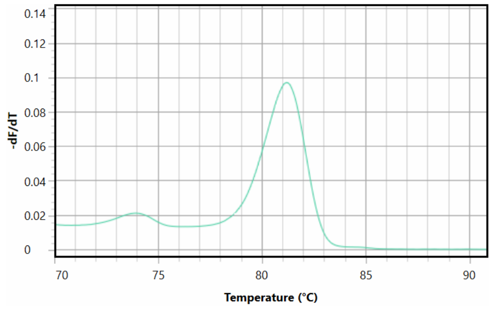

CqCPK29

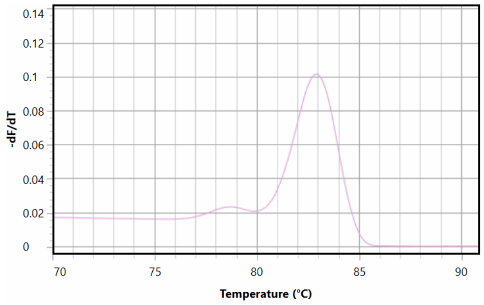

CqCPK32

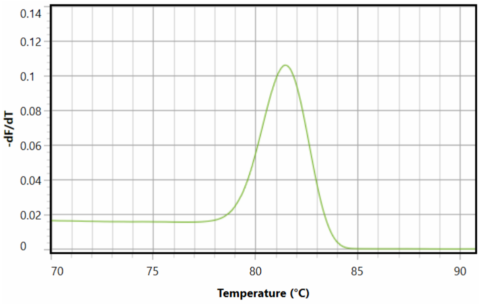

GAPDH-B

UNSA_VP033 - Leaves - 200mM

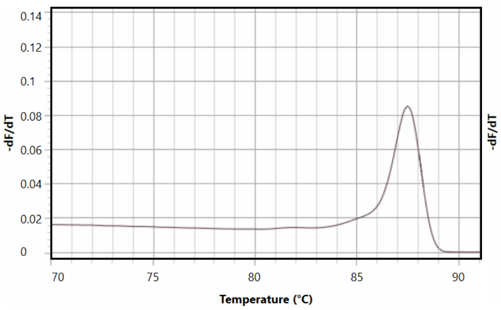

CqCPK3

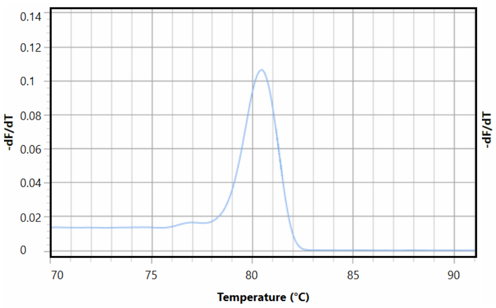

CqCPK4A

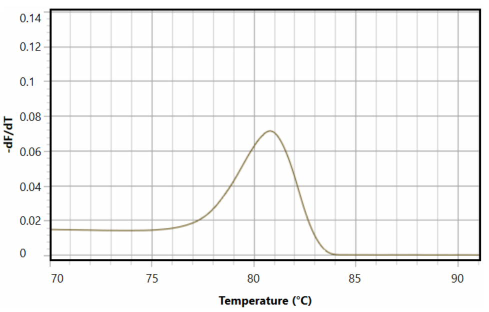

CqCPK4B

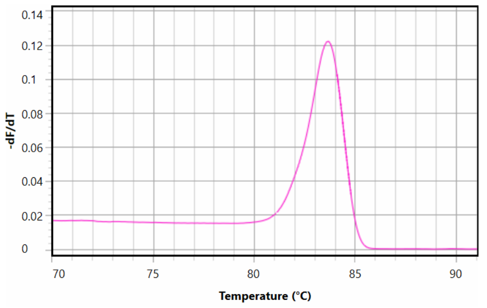

CqCPK9

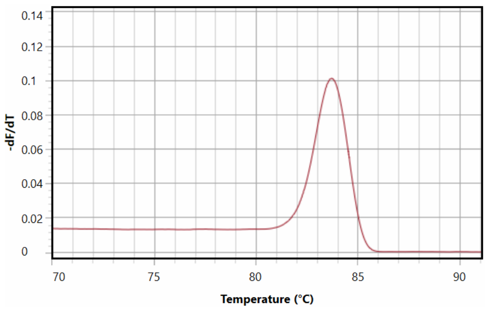

CqCPK10

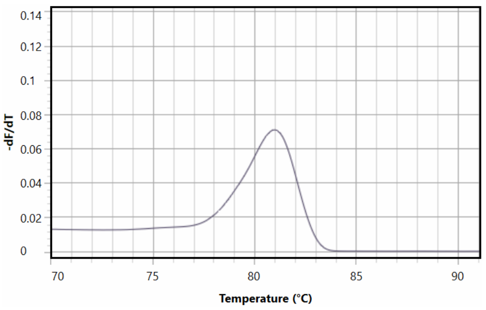

CqCPK12

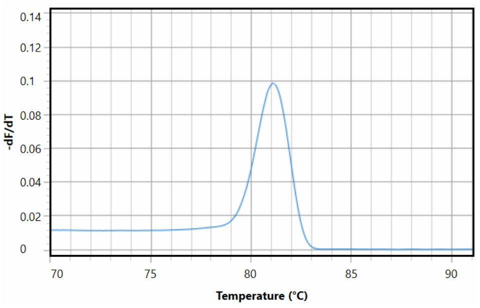

CqCPK16

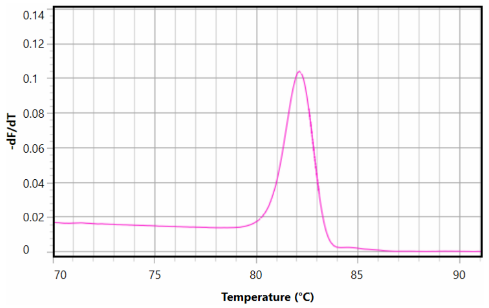

CqCPK17

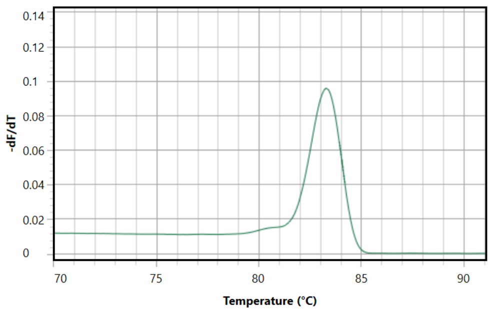

CqCPK20

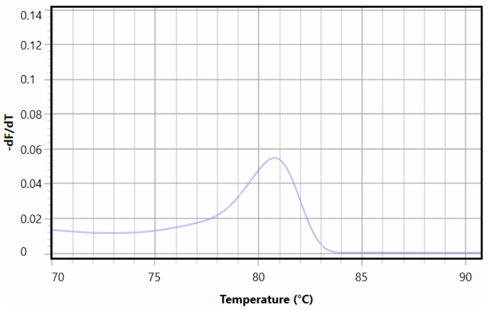

CqCPK28

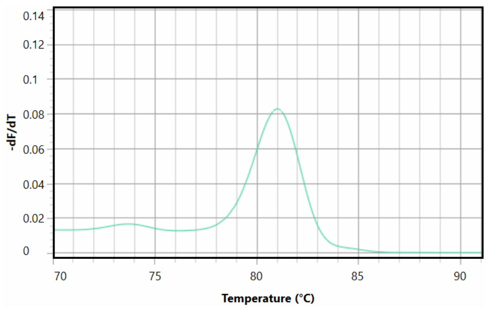

CqCPK29

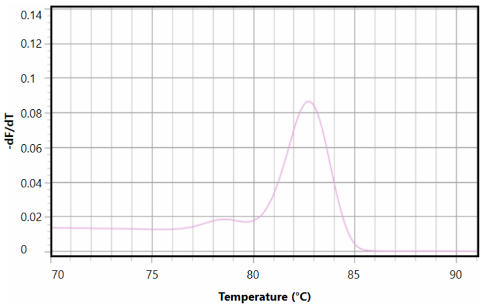

CqCPK32

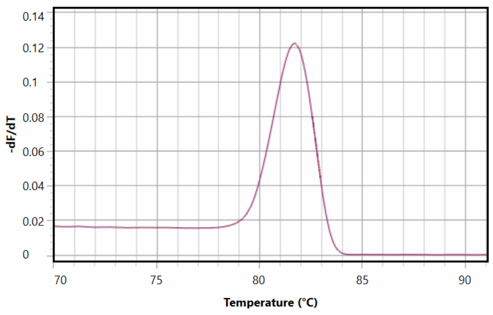

GAPDH-B

UNSA_VP033 - Leaves - 300mM

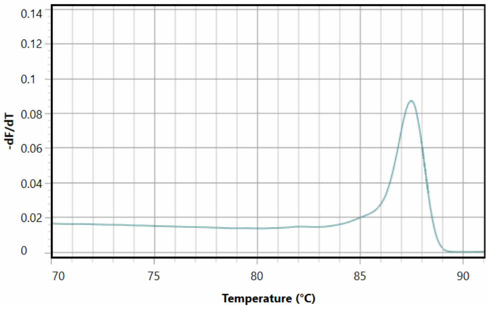

CqCPK3

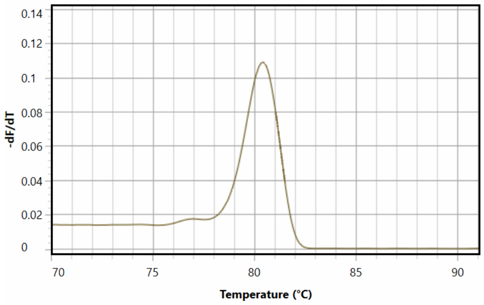

CqCPK4A

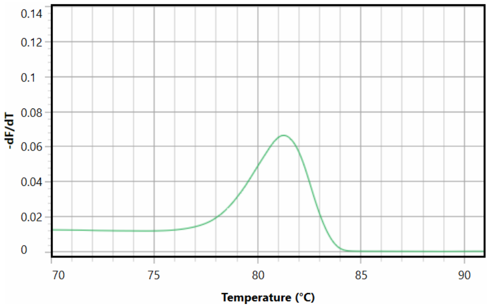

CqCPK4B

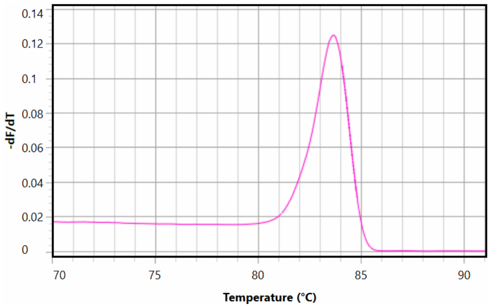

CqCPK9

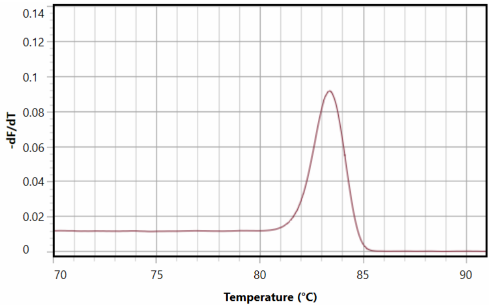

CqCPK10

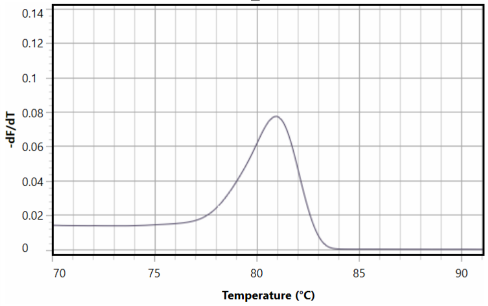

CqCPK12

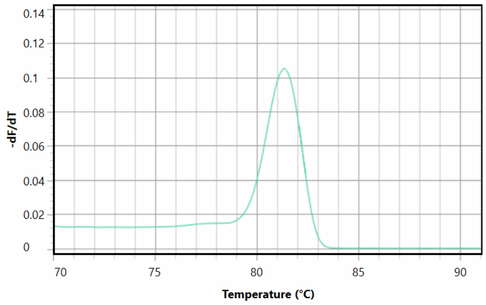

CqCPK16

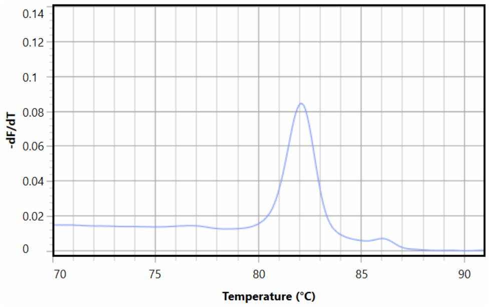

CqCPK17

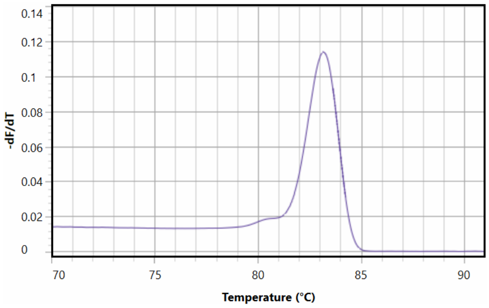

CqCPK20

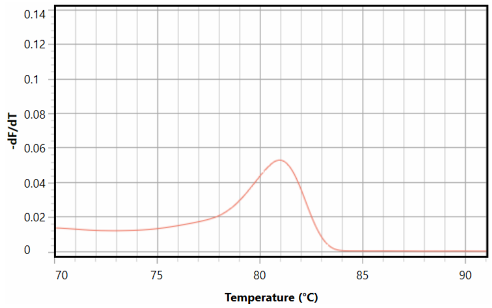

CqCPK28

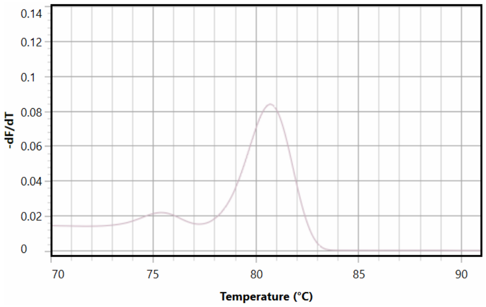

CqCPK29

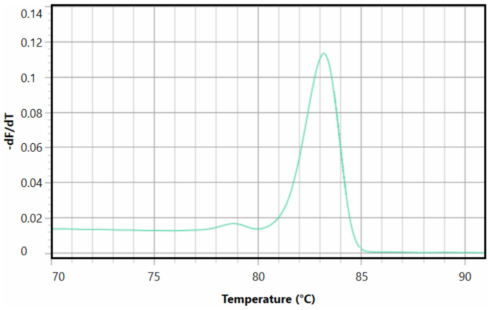

CqCPK32

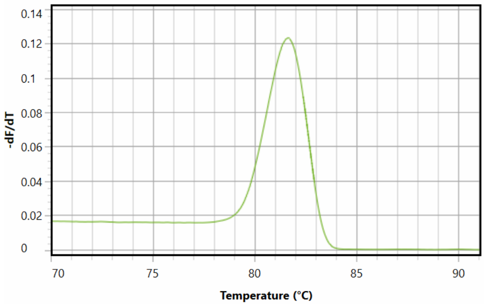

GAPDH-B

UNSA_VP021 - Leaves - 0mM

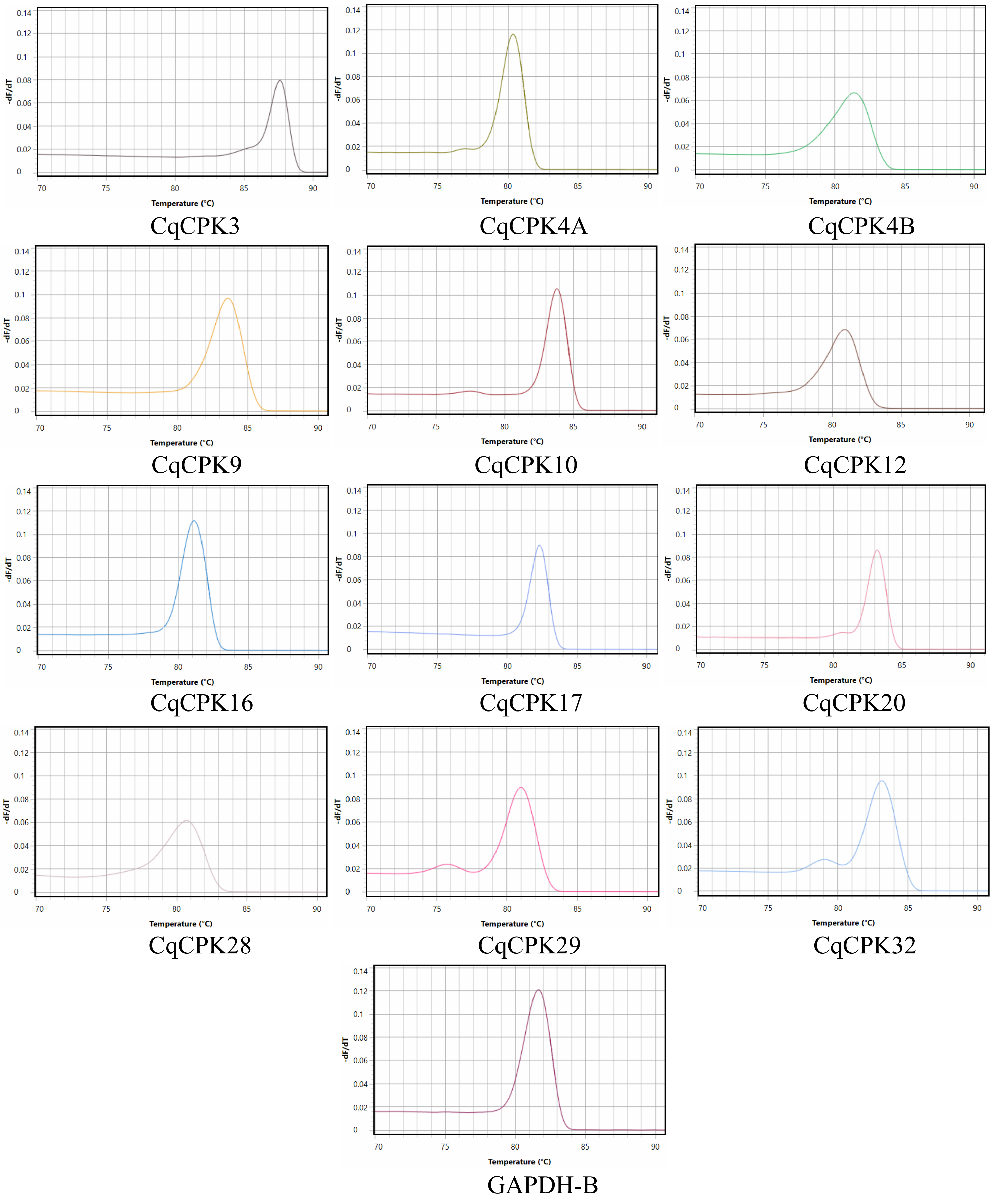

UNSA_VP021 - Leaves - 100mM

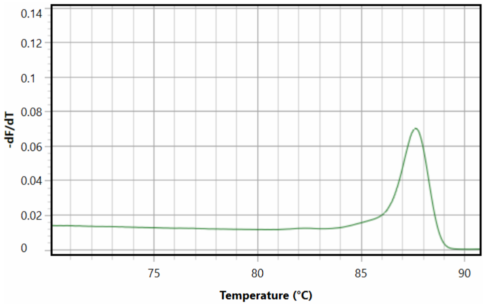

CqCPK3

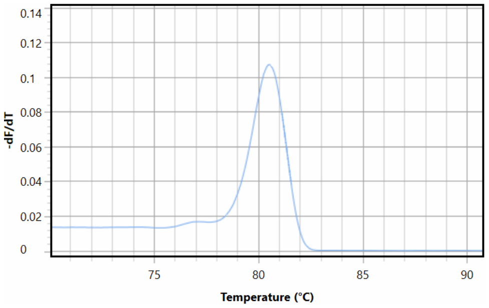

CqCPK4A

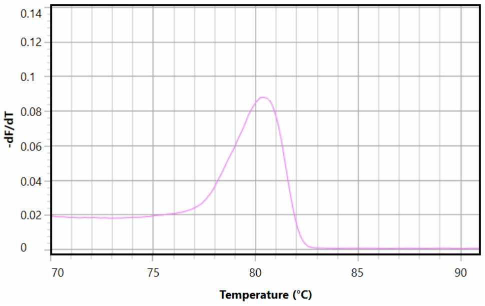

CqCPK4B

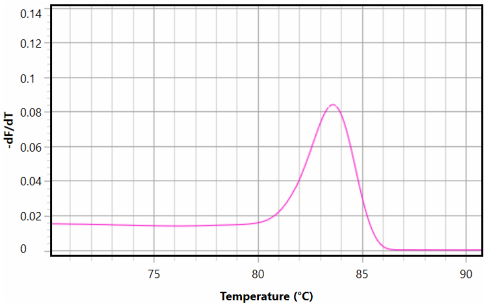

CqCPK9

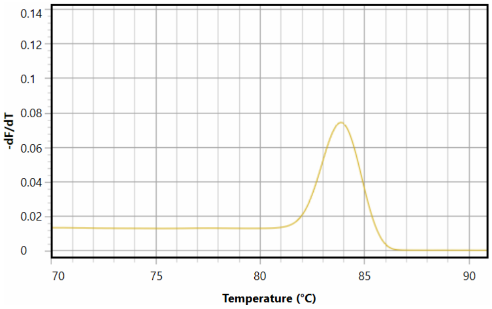

CqCPK10

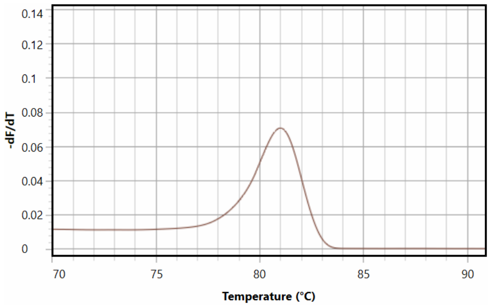

CqCPK12

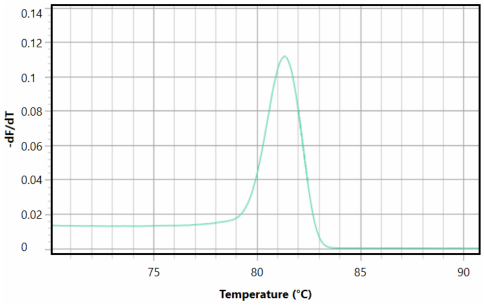

CqCPK16

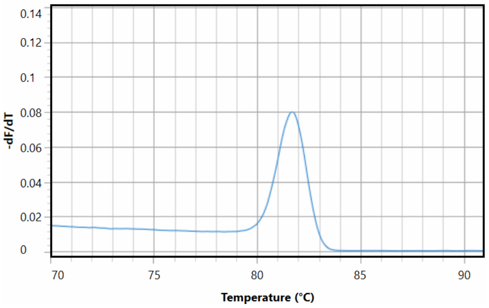

CqCPK17

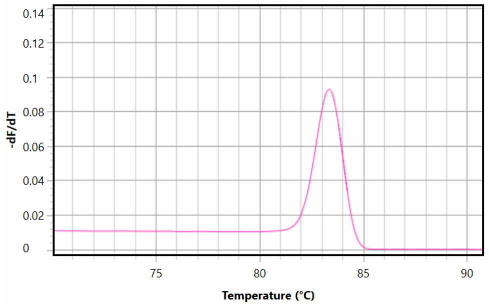

CqCPK20

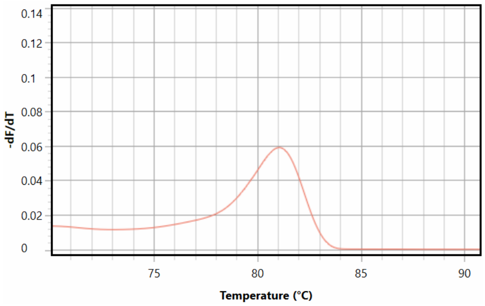

CqCPK28

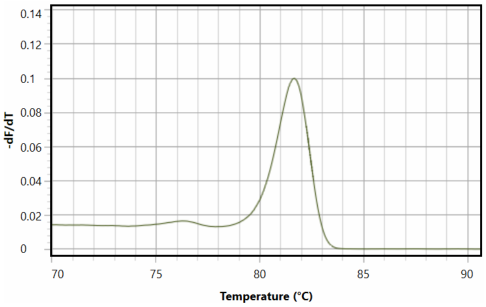

CqCPK29

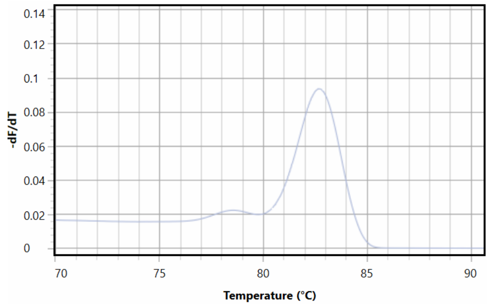

CqCPK32

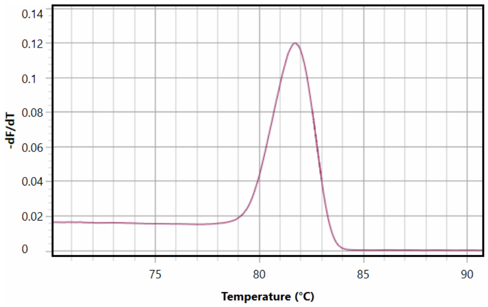

GAPDH-B

UNSA_VP021 - Leaves - 200mM

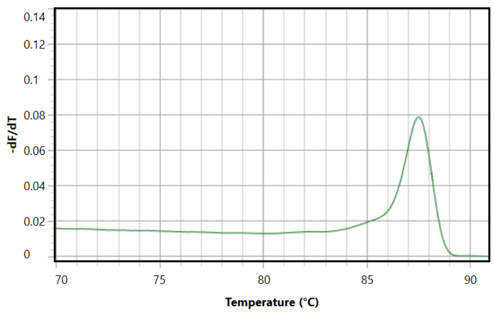

CqCPK3

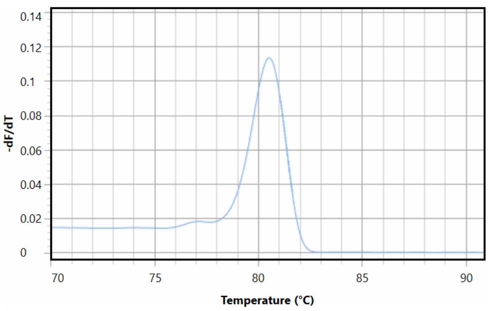

CqCPK4A

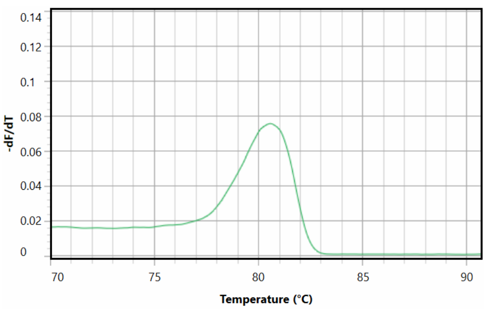

CqCPK4B

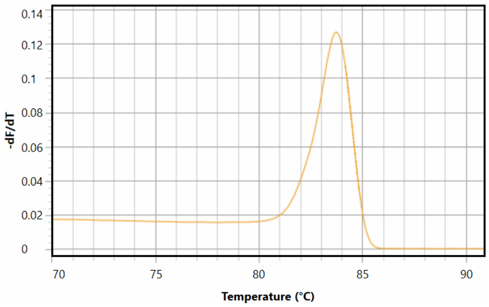

CqCPK9

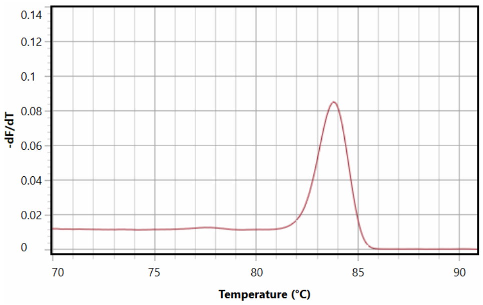

CqCPK10

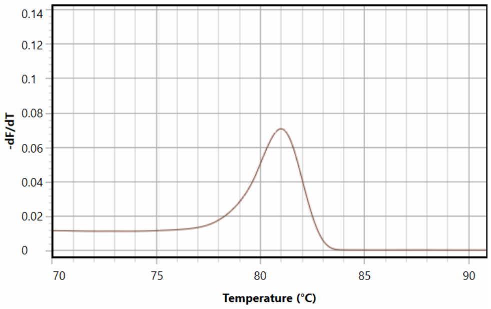

CqCPK12

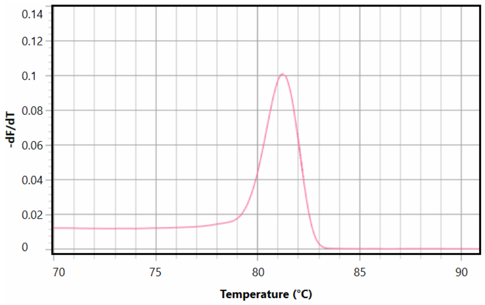

CqCPK16

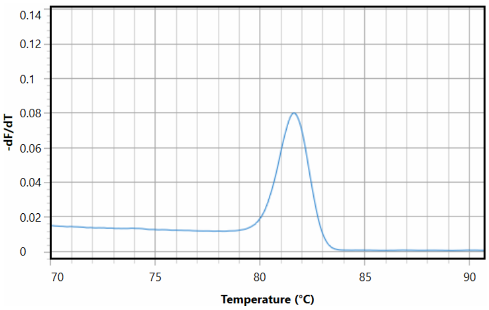

CqCPK17

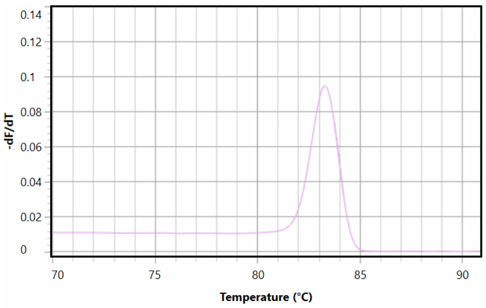

CqCPK20

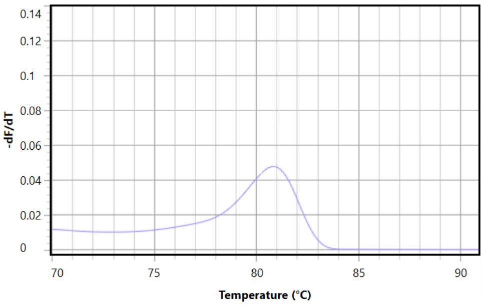

CqCPK28

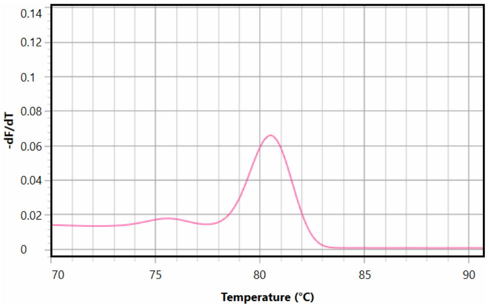

CqCPK29

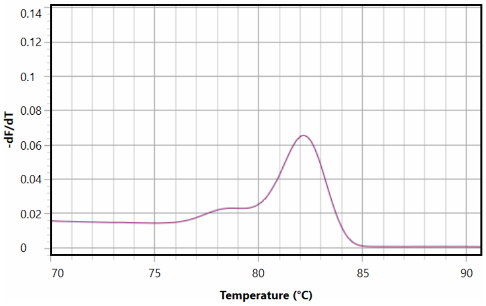

CqCPK32

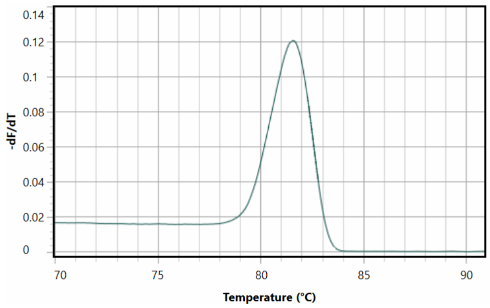

GAPDH-B
